# Process-based design of dynamical biological systems

**DOI:** 10.1038/srep34107

**Published:** 2016-09-30

**Authors:** Jovan Tanevski, Ljupčo Todorovski, Sašo Džeroski

**Affiliations:** 1 Jožef Stefan Institute, Ljubljana, Slovenia; 2 Jožef Stefan International Postgraduate School, Ljubljana, Slovenia; 3 University of Ljubljana, Slovenia

## Abstract

The computational design of dynamical systems is an important emerging task in synthetic biology. Given desired properties of the behaviour of a dynamical system, the task of design is to build an in-silico model of a system whose simulated be- haviour meets these properties. We introduce a new, process-based, design methodology for addressing this task. The new methodology combines a flexible process-based formalism for specifying the space of candidate designs with multi-objective optimization approaches for selecting the most appropriate among these candidates. We demonstrate that the methodology is general enough to both formulate and solve tasks of designing deterministic and stochastic systems, successfully reproducing plausible designs reported in previous studies and proposing new designs that meet the design criteria, but have not been previously considered.

Systems-based approaches to biology lead to better understanding of interactions in biological systems represented at different organizational levels. They rely on formalizing a model of a given system by specifying its constituents at a chosen organizational level, its structure, i.e. the interactions between the constituents, and the particular modelling assumptions for each interaction. The model is often used as a tool for analysis of the complex dynamical behaviour of the system over time and under changing internal and external conditions. Using models as analytical tools, we can obtain insights into the essential mechanisms that lead to emergence of complex dynamics in biological systems. In turn, these insights can be employed when solving the task of design, i.e. when constructing models of dynamical systems that exhibit a desired behaviour.

The most important input to the task of design is the knowledge about modelling dynamics in the domain of interest. This knowledge includes the systematic categorization of constituents of dynamical systems in the domain and the potential interactions between them. The second input to the design task are the design objectives, i.e. a set of expected properties of the desired dynamical behaviour of the system. The output of the design process is a candidate design (or a set thereof), i.e. a model with known structure and parameter values. To solve the design task specified above, we need to resolve two types of uncertainties. The first type of uncertainty is related to the model structure: The more different model structures we need to consider, the larger the uncertainty. To resolve the structural uncertainty, we need to select a model structure, i.e. to select a proper set of model constituents, the interactions among them, and make specific modelling assumptions on the kinetics of the interactions. The second type of uncertainty is related to the values of the constant parameters in the models, such as kinetic rates and initial conditions. We call this type of uncertainty parametric uncertainty. Resolving these two types of uncertainties leads to a candidate design (or a set thereof) that produces dynamical behaviour with expected properties.

In response to the increasing relevance of the task of designing biological systems for practical applications^1^,^2^, numerous computational approaches addressing the design task have been proposed. The approaches differ in the way the inputs and the uncertainties mentioned above are formalized and resolved. In particular, two classes of approaches are related to the work presented in this paper. The first class includes approaches that follow the “design by composition” paradigm, where valid compositions of standardized components with known types of interactions are sought for, given a design objective describing the relationship between designated inputs and outputs. The approaches in the second class follow the “design by optimization” paradigm, where the design objectives are transformed into objective functions that are then subject to optimization. This allows for considering a broader class of design objectives related to the qualitative and quantitative properties of the desired dynamical behaviour of the system. On the other hand, these approaches employ a rigid formalism for specifying the structural uncertainty that requires users to provide an explicit and complete equation-based specification of the structure of each candidate design.

Composition-oriented approaches are built upon the concepts of rule-based modelling and computer-aided design of electronic circuits. Rule-based modelling formalisms (and the related approaches to automated composition of parts), such as GEC^3^ and Eugene^4^, are used to describe constraints for the composition of a single design. These constraints are an addition to the domain knowledge specified in the form of a library of circuit components, based on standardised, well-characterized biological parts. Circuit components have fixed properties and rules define fixed parameter values for interactions among components. Thus, all parameters in a valid circuit have fixed values, which eliminates the parametric uncertainty. The structural uncertainty in these formalisms comes from the availability of interchangeable parts for the desired physical composition. However, these formalisms do not support automated resolution of the structural uncertainty. Instead, experts can use them to manually generate and test different valid compositions of biological circuits to achieve a given design objective. Composition-oriented approaches to automated resolution of structural uncertainty based on a design objective, such as Proto^5^ and Cello^6^ have been recently developed. The design objective in these approaches is formulated as a Boolean function of the defined inputs and outputs of the circuit. These approaches infer an abstract network representation of a composition of standard parts. The inference method is additionally constrained by the intended physical implementation of the circuit, i.e. the components that can be used to implement the intermediary logic functions (logic gates) needed to achieve the design objective. The final compositions produced by Cello and Proto can be resolved into a specific physical construct, i.e. DNA sequence of the composition using Eugene or more advanced methods, such as MatchMaker^7^. In any case, the application of these methods is limited to the specific task of designing biological circuits that realize a given transition function based on a single form of interactions between the connected components (represented by a sigmoidal transfer function).

In contrast, the approaches in the second class can handle more general design objectives, such as, for example, constrained values of the components of the Fourier spectrum of the system trajectory that are indicators of oscillatory system behaviour. However, they provide only simple tools for formalizing structural and parametric uncertainty by letting the user specify a list of candidate designs in the form of model equations with unknown parameter values. These approaches transform structural uncertainty into parametric uncertainty. ABC-SysBio^8^ requires the user to specify a list of equation-based models, each corresponding to a candidate design. It further reformulates structural uncertainty as parametric uncertainty by introducing an integer parameter whose value corresponds to the index of the candidate design in the list. Bayesian estimation methods are then employed to resolve the parametric uncertainty, producing a posterior distribution over the candidate model structures and the values of their parameters, which can be used for selecting designs that provide optimal fit to the design objectives. Other optimization-oriented approaches^9^,^10^,^11^,^12^,^13^ require the user to specify equation fragments and integrate them within a single equation structure using Boolean parameters that indicate the presence of individual fragments in the structure. The structural uncertainty is thus transformed into parametric uncertainty that is then resolved by using parameter estimation. In both cases, the formalization of domain knowledge and uncertainty is rigid and requires the users to directly encode complete mathematical models of design candidates. Some of these approaches transform multiple design objectives into a single objective function for optimization^9^,^10^,^12^, while others^11^,^8^,^13^ use multi-objective optimization methods that can consider multiple objectives simultaneously.

The central contribution of our work is a new approach to automated design that combines the flexibility of the design by composition approaches with the generality of the design by optimization paradigm. Our approach allows for flexible formalization of both structural and parametric uncertainty through a library of domain knowledge that specifies a taxonomy of design constituents and processes that describe their interaction composition. At the same time, the approach is able to handle a broad class of design objectives. In order to resolve the structural and parametric uncertainty, we bring together methods for combinatorial search and multi-objective optimization. The search space of candidate model structures is inferred from the specification of constituents and the potential interactions among them. The estimation of parameter values for each model structure employs a method for simultaneous optimization of multiple design objectives. The solution in each resulting Pareto front are aggregated by using a hypervolume based metric^14^, that is in turn used for the ranking and selection of candidate designs. Our approach builds upon the paradigm of process-based modelling^15^,^16^ that integrates formalized domain-specific knowledge and observed/measured data for automated modelling of dynamical systems. In the new setting, the design objectives, representing the desired properties of the behaviour of the system replace the objective of fitting the observed data. We adapt the process-based modelling paradigm to the design task where no measured/observed data are available. We conjecture that the newly proposed approach is capable of reconstructing the results of previous design efforts. In addition, we conjecture that the approach, due to the more flexible formalism for specifying domain knowledge and uncertainties, is also capable of discovering new promising designs not considered before. To test the validity of the two hypotheses, we apply the newly developed approach on two tasks with archetypical design objectives, i.e. designing a toggle switch and an oscillator. The first is based on a stochastic model of a genetic switch without cooperation that can be used as a basic memory unit^17^. The second is a deterministic oscillator based on a negative-feedback loop of protein interaction^18^.

## From process-based modelling to process-based design

Process-based modelling^15^ is an automated modelling paradigm that takes two inputs: formalized knowledge about modelling dynamical systems in the domain of interest and measurements of the observed system that is subject to modelling. The output is a set of models ranked according to how well they correspond to the observed system (i.e. fit the measured data about the system). The domain-specific knowledge about modelling is formalized as a library of entities (that represent constituents of the dynamical systems in the domain) and processes (that correspond to interactions between the entities). The knowledge library, when instantiated for a particular set of entities observed in a dynamical system at hand, provides a set of components for building models of the observed system. Process-based modelling approaches make use of combinatorial search to explore the space of candidate model structures that can be built from these components. The values of the parameters in these structures are estimated by using optimization methods that minimize the discrepancy between the measured system behaviour and the behaviour obtained by simulating the model. Following the search and optimization, process-based modelling approaches provide at output a list of models ranked with respect to their likelihood of reconstructing the observed system behaviour.

Process-based modelling has been successfully applied to a variety of modelling tasks in biology^19^,^20^,^16^ and other domains^21^. It has several advantages over other modelling paradigms that make it particularly suitable for adaptation to the task of design. First, process-based models retain the *understandability* and explanatory power of graphical model representations by providing clear insight into the structure of the observed system in terms of its constituents (entities) and interactions (processes) among them. Second, at the same time, they inherit the *utility* of mathematical models for simulation and analysis of system behaviour. Third, process-based models provide *general* model descriptions that support both stochastic and deterministic approaches to modelling, simulation and analysis. Finally, the knowledge representation formalism facilitates *modularity*: the knowledge library can be instantiated into a number of model components that are tailored to a particular modelling task at hand.

The most important and distinguishing aspect of process-based modelling is its ability to formally describe two different kinds of modelling uncertainty: uncertainty in the model structure and uncertainty in the model parameters. The structural uncertainty is captured in the formal description of domain knowledge (entities and processes) and is made explicit by transforming the latter into a space of candidate model structures (as opposed to the case of considering a single structure where we have no structural uncertainty). Process-based modelling approaches then employ combinatorial search methods to resolve the structural uncertainty. The very same methods can be used for process-based design. On the other hand, the parametric uncertainty is described by the formal specification of ranges of values for the model parameters. Given a model structure and measurements of the system behaviour, the values of these parameters are estimated by using standard optimization methods. The optimization objective functions (criteria) and the score for ranking the process-based models are derived from the following components:

C_1_ Measured behaviour of the observed dynamical system.

C_2_ Model behaviour obtained by simulation.

C_3_ Model complexity, in terms of model entities, processes and parameters.

The basic, most commonly used objective function, stems from the maximum likelihood principle, and uses C_1_ and C_2_. It measures the discrepancy between the measured system behaviour (*x*_*i*_) and the simulated model behaviour (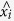),


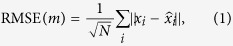


where *i* iterates over the observed system variables and *N* denotes the number of measurement time points. Another commonly used objective function relies on the parsimony principle that takes into account model complexity C_3_. If the complexity of the model is implicitly encoded by the values of the constant parameters, the objective function shown in equation (1) can be regularized by adding a component that takes into account the magnitude of the model parameters. A more general objective function can be obtained by following the minimum description length (MDL) principle^22^,


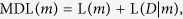


that takes into account L(*m*), the length of the minimal code necessary to completely encode the model (based on C_3_), and L(*D*|*m*), the length of the code describing the discrepancy between the simulated (C_2_) and measured behaviour (C_1_). The criteria based on the parsimony principle are useful when there is a need to distinguish between the suitability of multiple competing models with different structures.

For the design task, C_1_ cannot be used as a component of the objective function, since no measured data is available at input. For the design task, a second input is available that can replace the measured data:

C′_1_ Expected properties of the desired model behaviour.

Following this change, the RMSE criterion (Equation (1)) is replaced with one that combines C_1_′ and C_2_. A suitable design might have to fulfil multiple design objectives (expected properties), which (in general) can be independent or even conflicting. The discrepancy between each expected property of the system behaviour and the same property of the model simulation can be observed and used in the new criterion. Examples of multiple expected independent properties used for designing a system with oscillatory behaviour include the oscillation frequency and amplitude.

The issue of satisfying/optimizing multiple objectives can be addressed either by aggregating the corresponding objectives (i.e. the discrepancies between the expected and the actual value of the property of the behaviour) and using a single-objective optimization method or by simultaneous optimization of the objectives using a multi-objective optimization method. The aggregation of multiple objectives requires the introduction of a subjective weighting of the individual objectives in the aggregation. The subjective weighting can cause loss of information, especially in cases where independent or conflicting objectives guide the optimization. This makes a strong case for using multi-objective optimization methods to simultaneously handle multiple design objectives.

Finally, note that we do not use C_3_ as a component of the objective function for parameter estimation, since the complexity of the model structures in process-based modelling/design remains constant throughout the optimization of its parameter values. However, we take into account the component C_3_ in the final ranking of the models, when models with varying structural complexity are considered.

### Algorithm 1. Formal representation of modelling knowledge for protein binding


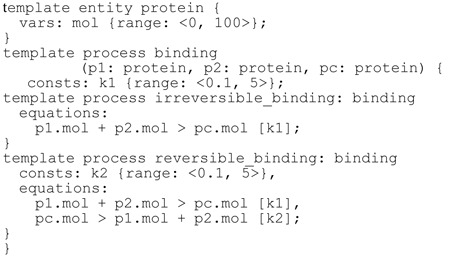


## Process-based design

We developed (designed and implemented) ProBMoTd, a tool for process-based design, as an extension of the process-based modelling tool ProBMoTs^16^. The extension proceeded in the following directions. First, we made use of our recent upgrade to the formalism for building process-based models from differential equations to reaction equations (also referred to as reaction networks), a formalism commonly used for stochastic and deterministic modelling in systems and synthetic biology^23^. Second, following the discussion from the previous section, we employed multi-objective (instead of single-objective) optimization methods for solving the parameter estimation task. Finally, we introduced a new model-selection score for ranking the candidate models that takes into account the design objectives and the complexity of the model structure.

Following the process-based modelling paradigm, the task of process-based design takes as input a library of knowledge about system constituents and interactions in the domain of interest, encoded by using template entities and processes. An entity represents a constituent of an observed system with its constant and variable properties. For instance, in a simple model of a system that involves protein binding, an entity corresponds to a protein with a single variable property mol denoting the number of its molecules present in the system. A formal description of a template entity corresponding to a protein is given at the top of Algorithm 1: this template can be instantiated to multiple different proteins that need to be considered for a specific dynamical system.

Similarly, in this example, processes describe the binding interaction between protein entities. The first template process in Algorithm 1 corresponds to an abstract binding interaction, while the following two template processes represent two more specific types of binding: irreversible and reversible binding. The template process 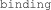 specifies that a binding interaction involves three constituent entities, all of the same type — 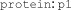 and 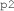 denote the binding proteins and 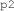 denotes the protein complex resulting from the binding process. The two subordinate processes 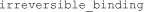 and 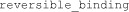 inherit the involved entity attributes (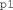, p2, pc) as well as the constant parameter corresponding to the binding rate (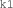) from their parent (the template process 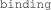). Each of the subordinate binding processes specifies the final template reaction equations used to model the binding interactions; for a particular system being modelled, only one of these two alternatives with specific values of the constant parameters will apply.

Given a specific system with three proteins 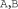 and 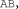, an incomplete model can be specified that contains a single process instance 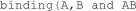. Note that, by this specific instantiation, the incomplete model formalizes the structural uncertainty: it defines a space of two candidate model structures, one containing a process of irreversible and the other a process of reversible binding. While each of these structures contains a different form of the binding process the values of the constant parameters 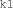 and/or 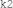 remain to be estimated in both; in other words, besides structural, we also have parametric uncertainty. To resolve it, we employ parameter estimation, where the parameter values are optimized with respect to the design objectives, i.e. the expected properties of the desired system behaviour. An example objective can aim at a specific steady-state of the system. In particular, we observe the property of the behaviour of the system S(*x*) that corresponds to the number of molecules of *x* when the system reaches a steady state. A possible formulation of the design objective is that 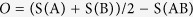 comes as close to the target value of 0 as possible. The parameter estimation will find optimal values of the model parameters for each of the two candidate models. The optimal value of the objective would indicate the suitability of the model candidate. In this simple example, the reversible binding is expected to be a more suitable alternative for achieving the selected objective.

In general, however, design tasks include a number of objectives corresponding to different expected properties of the desired behaviour. In order to support multi-objective parameter estimation, we integrated within ProBMoTd the implementation of Generalized Differential Evolution^24^ from the Java-based framework for multi-objective optimization^25^. In contrast to a single optimal point obtained in the case of a single objective, the result of multi-objective parameter estimation (for a given model structure) is a set of Pareto-optimal points from the parameter space (referred to as the Pareto front) together with the corresponding values for each objective. To rank the candidate model structures, we ranked the corresponding Pareto fronts. To this end, we calculated the hyper-volume under each of the Pareto fronts, HVUPF^14^, i.e. the volume between the set of points on the Pareto front and the origin point (that corresponds to the optimal values of the objectives)^26^.

To use HVUPF as a score for ranking the candidates, several assumptions (that do not limit the applicability of the approach), should be met. First, each objective should have a finite domain of possible values that is known a-priori. Second, all the objectives should be formulated in a manner that requires their minimization. Under these assumptions, a candidate model structure with a smaller HVUPF outperforms the candidates with larger volumes. A simple design selection strategy is to choose the model with the smallest HVUPF. However, as in the case of modelling, these estimates can be biased towards more complex models. To address this issue, we introduced a selection score that penalizes complex model structures, where complexity is measured as the number of reaction equations. For finite spaces of candidate model structures, *M*, both HVUPF and the model structure complexity were normalized to the [0, 1] interval and combined in an MDL-like score for a single model structure *m* as follows:


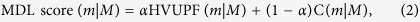


where *α* is a parameter in the interval [0, 1] used to trade-off between the HVUPF and the model structure complexity (C). At output, ProBMoTd reports the list of the candidate models ranked with respect to the descending value of the score. When reporting the results of the empirical evaluation, we visualized the score profile of the ProBMoTd output as a bar plot, where x- and y-axes correspond to the candidate model ranks and the model scores (on a logarithmic scale), respectively.

## Results

### Methodological contribution

Before reporting the results of the empirical evaluation of the proposed approach (in terms of its ability to reconstruct known results of previous design efforts and propose new designs), we summarize the methodological contribution of the paper and restate its position within the context of related approaches to automated design. In particular, we present the workflow used to formalize and resolve structural and parametric uncertainties with the process-based design approach.

**Figure 1:**
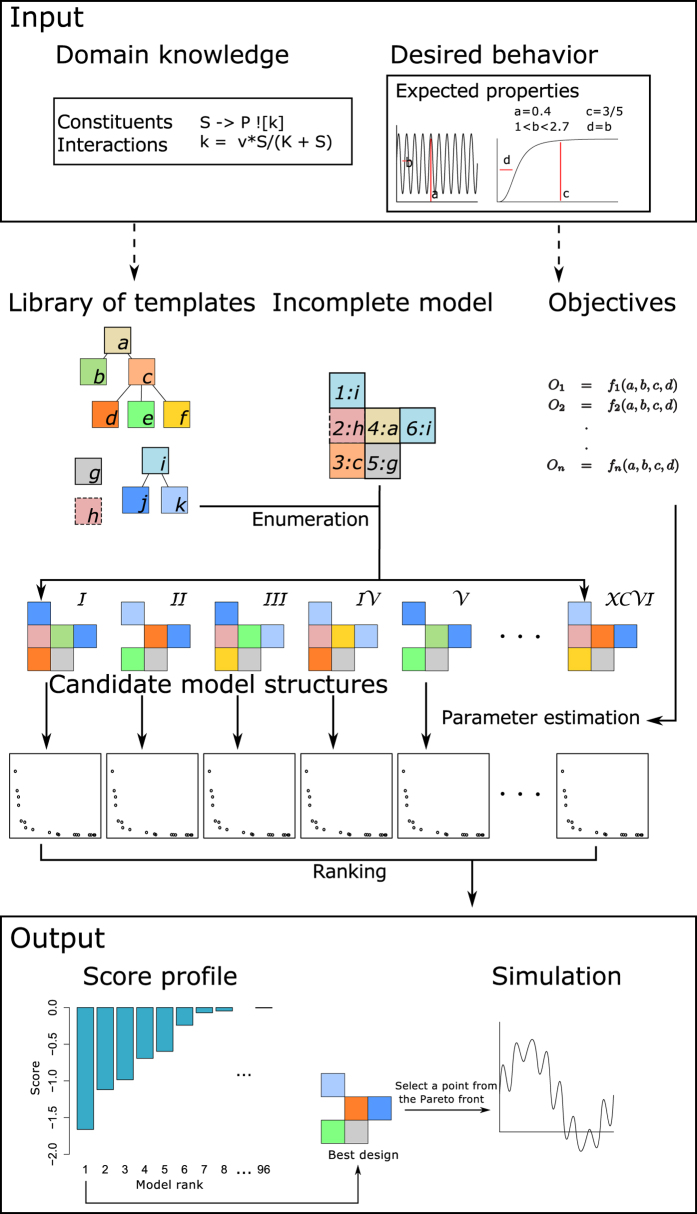
The workflow of our process-based design approach. At input, it takes domain-specific knowledge about modelling systems in the study domain and expected properties of the desired behaviour. The knowledge is formalized as a taxonomy of modelling templates and a specification of an incomplete model. The incomplete model uses inner nodes in the taxonomy to specify a set of alternative design choices; during the enumeration of the candidate model designs, these are instantiated with leaf nodes in the taxonomy that correspond to specific design choices. The parameters of each candidate model are estimated using multi-objective optimization with objectives corresponding to the expected properties of the desired system behaviour, yielding a Pareto front of solutions for each candidate. Finally, at output, the candidate designs (model structures) are ranked according to the hyper-volumes under their Pareto fronts obtained with multi-objective optimization. For each design, the output contains its structure, parameters and simulated behaviour.

Figure 1 recapitulates the workflow of the process-based design approach that was introduced in the previous section. Given the two inputs to the design task, the domain-specific modelling knowledge and the expected properties of the desired behaviour, an expert has to prepare the input to ProBMoTd. First, following the composition-oriented approach to design, the domain knowledge is encoded in a library in the form of a taxonomy of template entities and processes that can be used for modelling any system in the domain at hand. Next, for the particular design task, constraints are specified on how entity and process templates from the library of domain knowledge can be instantiated and composed into candidate designs. This is done by the specification of an incomplete model, which formalizes the structural uncertainty. To resolve the structural uncertainty, ProBMoTd enumerates the candidate model structures by combining the incomplete model specification with the library of entity and process templates. Components of the incomplete model that correspond to the inner nodes of the template taxonomy are instantiated with their subordinate leaves, leading to multiple candidate model structures. For example, position *1* in the incomplete model can be replaced by instances of two alternatives (*j* and *k*; see the incomplete model and library of templates visualization in Fig. 1). The instance of the alternative (*j*) appears in the candidate structures *I*, *III* and *V*. Components with dashed borders (such as component *h*) are optional. Position *2* in the incomplete model can thus either be instantiated using the template *h* (as in model structures *I*, *III*, *IV*) or omitted (as in model structures *II* and *V*).

The design objectives, i.e. the expected properties of the desired system behaviour, are reformulated as objective functions for optimization (following the optimization-oriented approaches to design). For each candidate model structure, a multi-objective optimization method is used to fit the parameter values (so as to minimize the objective functions) resulting in a Pareto front of sets of parameter estimates and their corresponding values for the objective functions. In turn, the HVUPF score is used to aggregate the values of the objective functions for the points on the Pareto front into a single score of the design that is used to rank the candidates (equation (2)). Experts can then analyse the ProBMoTd output, i.e. the score profile of the (top ranked) candidate designs and select some of them for further exploration. To obtain a simulation for a selected candidate design, the user has to select a single point from the Pareto front: the points on the Pareto front are by definition such that none of them is better/worse than all of the others on all design objectives. Note, however, that the decision making requirement for the user has been maximally postponed to the point where complete information is available about the best design structure and its possible parametrizations. This information characterizes the design’s ability to achieve the desired behaviour and allows the user to make an informed choice. By default, it may be useful to select a point from the Pareto front positioned in a region in the objective space that is as close as possible to the origin point, for which the design can be considered to achieve satisfactory behaviour, but the user may select alternative points from the Pareto front based on their preferences.

### Stochastic toggle switch without cooperativity

The synthetic toggle switch^27^ is one of the first synthetically designed systems that can achieve bistable switch-like behaviour. The importance of a simple synthetic switch is its potential use as a basic memory unit able to hold one bit of information. Its simple design contains two genes coding for proteins that mutually inhibit their production. The system can be controlled by inducer molecules that change the steady state of the system from a state where one protein has a low number of molecules while the other has high to the opposite one. In our work, we approached the task of designing a toggle switch without cooperative binding. Lipshtat *et al*.^17^ showed that the basic toggle switch without cooperativity might not always be able to achieve a switching behaviour, due to the possibility of a deadlock state where both the number of proteins A and B in the system is zero. They proposed different mechanisms to improve the design in order to achieve a more robust switch like behaviour. In a later study, Barnes *et al*.^8^ considered the task of selecting a most suitable model among four candidates, which contain one of the proposed mechanisms by using a Bayesian approach. In both studies, the candidate model structures were explicitly and manually enumerated.

We next describe in detail the process of preparing the input to the task of process-based design, i.e. the library of templates and the incomplete model, which uses domain knowledge from the previous studies. The formal representations of the library of domain knowledge and the incomplete model are shown in Supplementary Table S1 and Supplementary Table S2, respectively. The system is composed of constituents that we described using five template entities: 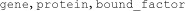 (describing a protein bound to the promoter region of a gene), 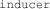 (describing an external inducer controlling the number of molecules of a specific protein in the system) and 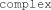 (describing a complex formed by binding of an inducer to a protein). Each template entity has a single variable property 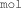, which represents the number of molecules of the corresponding entity present in the system in a given state at a given time. Additionally, in the template entity 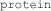, we defined two constant properties (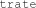 and 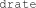) which correspond to the rates of production of a protein from its corresponding gene (accounting for both transcription and translation) and the rate of its degradation.

**Figure 2:**
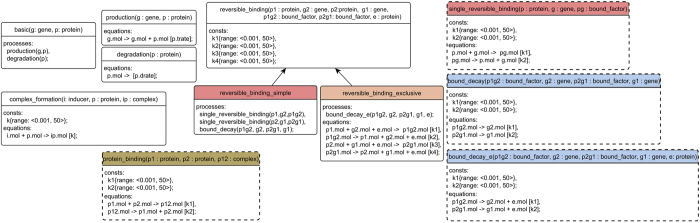
Taxonomy of template processes included in the library of domain knowledge for designing a stochastic toggle switch. The arrows in the taxonomy point from the child process template to the parent process template. The template process properties include a list of nested processes (components of the process in which they are nested), constants (usually corresponding to local kinetic rates) and equations (list of reaction equations describing the process). The template processes defined in a box with a dashed border represent optional processes.

Figure 2 depicts the taxonomy of all template processes used to describe the (possible) interactions within the system. To represent the basic processes for a single gene coding for a protein we specified the template process basic. This template contains two subprocesses, a process of 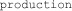 of a protein product from its coding gene in an unbound state, and a process of protein 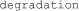. Furthermore, we defined a template process (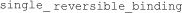) describing a single reversible binding of a protein to the promoter region of a gene, forming a bound factor complex and thus inhibiting the production of the protein which the bound gene codes for. We made this template process optional: An optional template process represents a two level hierarchy in which the top-level template process has two subordinate (child) template processes; one describing an empty process (representing the absence of interaction) and the other representing the presence of interaction. To account for the function of the inducer, we defined a template process 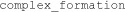 which describes the irreversible binding of an inducer with a free protein and the formation of an inducer-protein complex.

In the library of domain knowledge, we next introduced the mechanisms suggested by Lipshtat *et al*.^17^ as alternative or optional processes. First, we introduced an alternative to the simple reversible binding that allows for modelling exclusive binding (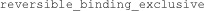). The exclusive binding assumes that both genes share a single binding site. Following Barnes *et al*.^8^, we introduced in the template process a protein entity e to which a protein should bind to before binding to the corresponding gene. Second, we introduced an optional template process describing reversible 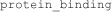 of repressor proteins. Finally, we introduced two optional template processes that describe the degradation of a repressor protein while bound to the gene (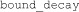 and 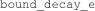). These optional template processes define 2 modelling alternatives each, like the protein-protein binding template process.

**Figure 3:**
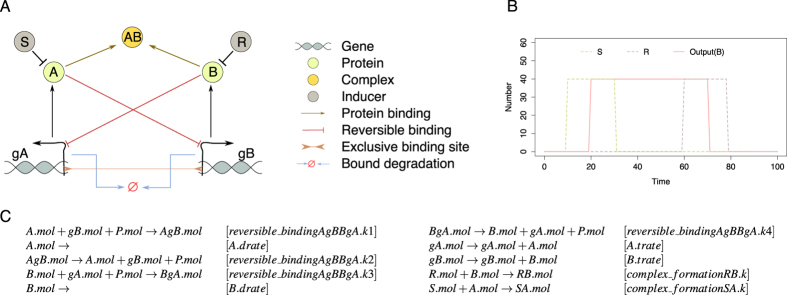
(**A**) Graphical representation of the toggle-switch interaction structure composed of instantiated templates from the library of domain knowledge. The colors of the instantiated components correspond to the color of the template processes from Fig. 2(B) The expected behaviour of the observed output variable (number of molecules of protein (**B**) and the input provided to the system (number of molecules of the inducers S and R) as a function of time. (**C**) The instantiated reaction equations for a single candidate design corresponding to the exclusive switch, where the symbol in the rectangular brackets denotes the rate (constant parameter) of the corresponding reaction.

Given the basic structure of the toggle switch, we were interested to find whether it has the optimally complex structure needed for achieving switching behaviour. Therefore, we explored the possibility of a simpler model structure (presence/absence of inhibitory interactions). An additional point of interest, which has not been addressed previously, is the possibility of achieving a better switch-like behaviour by considering combinations of the proposed mechanisms. To this end, we instantiated the specific constituent entities and processes as depicted in Fig. 3A. We instantiated two genes gA and gB from the template entity gene, two proteins A and B, two inducers S and R, the bound factors AgB and BgA, and the complexes SA, RB and AB.

The instantiation of the 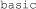 and the 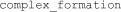 template processes, for the gene-protein pairs gA-A and gB-B, and for the inducer-protein pairs S-A and R-B respectively, describes only one possible alternative of interaction. The instantiation of the top-level 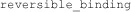 template process leads to 10 different alternatives of the process. Its mutually exclusive child template processes 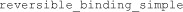 and 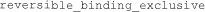 describe 2 × 2 × 2 = 8 and 2 alternatives correspondingly by their nested subprocesses: The subprocesses 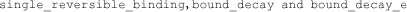 are optional and describe 2 alternatives each. The final instantiated top-level template process is the 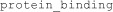 optional process (2 alternatives) which finally led to definition of a space of 1 × 1 × 1 × 1 × 10 × 2 = 20 possible candidate model structures. By taking advantage of the knowledge available in the library, these 20 candidate model structures include, in addition to the four candidate models manually enumerated in the previous experiments, 16 other viable alternatives with simpler or more complex structure, containing some or all of the suggested mechanisms for achieving a switch-like behaviour. The reaction equations for the exclusive switch are shown in Fig. 3C, while the reaction equations for all candidate design structures are presented in the Supplementary Section 2.2.

Our experimental setup built on the experimental setups defined in previous work. We interpreted each model stochastically, i.e. the network of reaction equations with stochastic kinetic rates was automatically transformed into a continuous-time Markov chain with a finite state space. During each step of the optimization process for each model, we performed 100 realizations in the time frame 0 ≤ *t* ≤ 100, sampled at each integer time point, which we used to calculate the values of the objective functions more accurately. The objective functions guiding the multi-objective optimization were defined as:


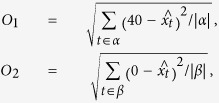


where 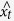 is the average simulated number of protein B molecules at time *t*, 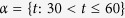, 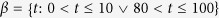, and 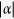 and 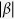 are the lengths of the intervals *α* and *β*. A model with optimal values for the objective functions (*O*1 = 0, *O*2 = 0) would have an ideal expected behaviour (as shown in Fig. 3B). Each candidate model structure has different complexity (number of reactions), which ranges from 6 for the least complex to 14 for the most complex structure. Therefore, the score used for ranking of each model structure was obtained by using the function from equation (2).

**Figure 4:**
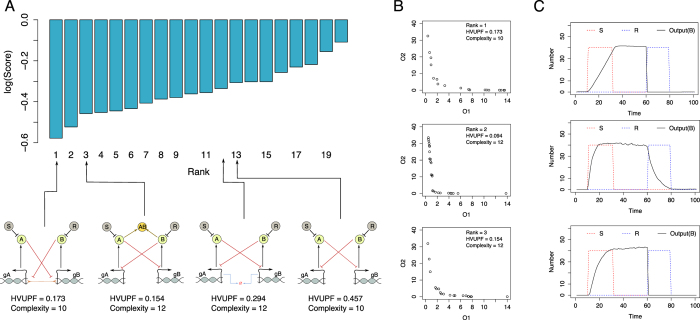
(**A**) Score profile containing all model structures ranked from the most appropriate to the least appropriate, for the stochastic toggle switch without cooperativity, according to the logarithm of the obtained score that takes into account complexity (lower score is better) (top). Sample candidate models containing structures considered in previous studies: from left to right according to their ranking, exclusive switch, switch with a protein-protein interaction, switch with bound repressor degradation, basic switch (bottom). (**B**) Pareto fronts of the three best performing models. (**C**) Simulations of the observed output variable (average of 100 realizations) of the three best performing models (using an arbitrarily selected point from the Pareto front).

Figure 4A shows the obtained score profile. Considering the ranking of only the four candidate model structures considered in previous studies (bottom of Fig. 4A), the best ranked candidate (rank 1) has a structure containing an exclusive switch, the second best ranked candidate (rank 3) has a structure containing a protein-protein interaction, while the toggle switch with bound degradation and the original toggle switch have similar performance (being ranked 12 and 13, respectively). Our relative ranking of the four designs corresponds to the ranking obtained by Barnes *et al*.^8^ and confirms the validity of our approach.

We further analysed the ranking and the structures of the models that perform better than the original toggle switch in relation to the aforementioned four models. Overall, the model structure containing an exclusive switch is ranked first, while the model structure with a protein-protein interaction is ranked third. Ranked second is the model structure containing both an exclusive switch and a protein-protein interaction, a model that has previously not been considered as a possible candidate model that can achieve the expected behaviour. It is worth noting that this model performs best in terms of HVUPF. The obtained Pareto fronts and simulations of these three models are shown in Fig. 4B,C. The model with bound degradation and the original toggle switch are ranked 12th and 13th. The Pareto fronts and simulations of the latter models are shown in Supplementary Figs S1–S2.

Other than the fourth ranked model that contains exclusive binding and bound degradation, in between (rank 5 to 12) we observed models that could be structurally separated into two clusters, i.e. a cluster of model structures that contain a protein-protein interaction and a cluster of model structures that contain processes of bound degradation. Considering the better ranking of the model structure containing a protein-protein interaction, we noticed that this mechanism represents a good alternative to the inhibition by protein-gene binding (regarding the ability of the system to achieve toggle switch behaviour). Additionally, in both clusters we observed structures that contain only inhibition of the production of protein B by binding protein A to gene gB. This is due to the experimental design in which we defined an expected property of the behaviour that is dependent only on the number of protein B molecules observed in the system.

### Robust negative feedback oscillations

Systems that can achieve stable oscillating behaviour are basic control parts that are critical for many biological systems. Therefore, the study of such systems, their improvement and implementation is an important task. Tsai *et al*.^18^ studied a small set of five oscillating networks, based on a design consisting of three proteins and a negative feedback loop. The main point of interest there was whether adding a single auto interaction will lead to improved robustness of the system. In the study, the robustness of a candidate model was defined by its operational range of frequencies. The operational range was established by first taking a limited sample of the space of parameter values for each candidate model, then examining the frequency and the amplitude of the oscillations (if oscillation was achieved) for each set of parameter values and finally calculating the differences of the minimal and maximal achieved frequency. Given the high nonlinearity of the models, the relationship between the space of parameters and behaviours is complex. Consequently, a sampling approach might not accurately approximate the operational range.

In a follow up study, using a Bayesian design approach, Barnes *et al*.^8^ focused on selecting a design out of five available candidates that can most likely achieve a fixed frequency and point-to-point amplitude. The Bayesian design approach considers concurrently different objective functions that guide the Markov chain Monte Carlo sampling process used to establish posteriors over the parameter values and model structures. However, it is computationally very demanding and therefore not feasible for use in the wide range of experiments that need to be performed to establish the robustness of oscillatory behaviour.

**Figure 5:**
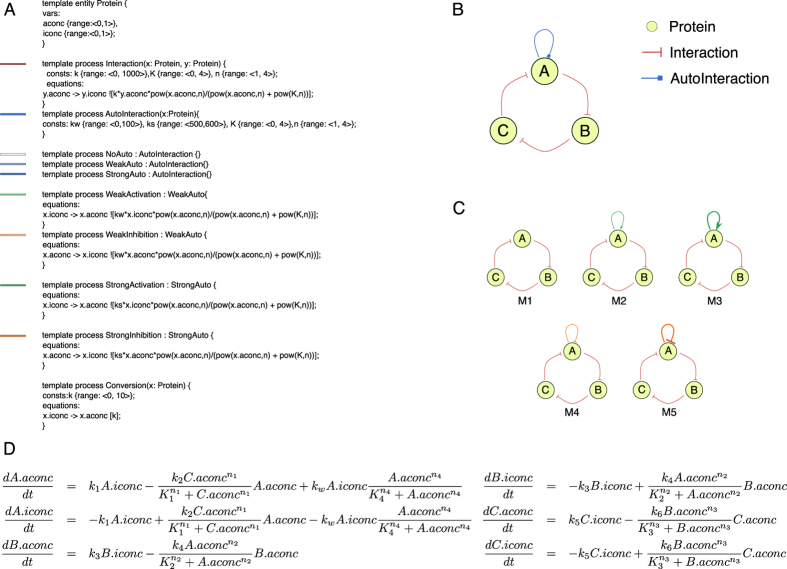
(**A**) The library of domain knowledge used for designing systems with robust negative feedback oscillations encoded in the process-based formalism (template entity and processes). (**B**) Graphical representation of the incomplete structure of the models as instantiated using components from the library of domain knowledge. (**C**) The five candidate model structures enumerated by the incomplete model. The thin loops in models 2 and 4 represent weak auto-interaction while the thicker lines in models 3 and 5 represent strong auto-interaction. The green lines represent auto-activation, while the orange lines represent auto-inhibition. (**D**) The system of coupled ordinary differential equations for the candidate design structure M2.

As shown in Fig. 5A, we encoded the knowledge available from the aforementioned studies into a library of domain knowledge by using the process-based formalism. In order to model a negative feedback loop of interacting proteins, we first introduced a template entity representing a protein with two variable properties: active concentration and passive concentration. We next introduced two top level processes: 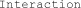 and 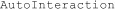. The former describes a directed interaction between two protein entities. For modelling a negative feedback loop, we required only an inhibition interaction between two entities. Hence, we described the interaction as a change of the form of the protein (affected by the inhibition) from active to inactive, following a Michaelis-Menten rate law with cooperation (catalysed by the active form of the affecting protein). The latter was also used to describe an interaction loop for a single entity. In order to encode all possibilities as described by Tsai *et al*.^18^, we defined a taxonomy of template processes. The 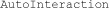 template process is inherited by three second level template processes: 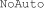, which describes the case where there is no interaction loop; 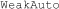, which describes a weak interaction; and 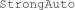, which describes a strong interaction. The 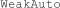 and 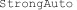 template processes are inherited by leaf template processes which describe the corresponding weak and strong activation and inhibition interactions.

Using the described library of domain knowledge, we were able to define an incomplete model that can be refined to obtain all five candidate model structures described by Tsai *et al*.^18^. The incomplete model is graphically depicted in Fig. 5B. The formal representation of the incomplete model is shown in Supplementary Table S3. We instantiated the template entity into three protein entities A, B and C. We defined an inhibitory loop by instantiating the 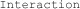 template process into the three required inhibitory interactions. Finally, we instantiated the top-level 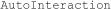 template process with protein A as its argument, effectively defining the space of possible candidate model structures. The five model structures described by the incomplete model are depicted in Fig. 5C. The system of coupled ordinary differential equations for the candidate design structure M2 is presented in Fig. 5D; the differential equations for the other four candidate designs are presented in the Supplementary Section 3.2.

To evaluate the performance of our approach and (at the same time) establish the most robust negative feedback oscillator structure (in terms of its ability to achieve oscillations over a range of exact frequency - amplitude pairs), we performed two sets of nine design tasks. In order to compare our results to those of the previous studies, we considered (as target expected properties of the behaviour) the frequency-amplitude pairs formed by the Cartesian product of frequencies *f*_*t*_ = {0.1, 1, 10} and amplitudes *A*_*t*_ = {0.01, 0.1, 1}, for the active concentrations of protein A and C (protein B is included by symmetry). Each candidate model was interpreted deterministically by automatically transforming the set of reaction equations to a system of ordinary differential equations, followed by a simulation in the time frame 0 ≤ *t* ≤ 28 (sampled with a frequency of 40 Hz). The objective functions guiding the multi-objective optimization were defined as:


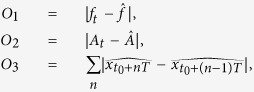


where *f*_*t*_ is the target frequency, 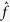 is the frequency obtained by calculating the largest component of the Fourier spectrum of the simulated trajectory of the model, *A*_*t*_ is the target amplitude, 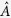 is the amplitude determined from the simulated trajectory of the model, 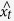 is the simulated active concentration of the target protein *x* at time *t*, 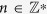 and 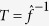. All values were calculated using *t*_0_ = 2*s* in order to remove initial transients.

**Figure 6:**
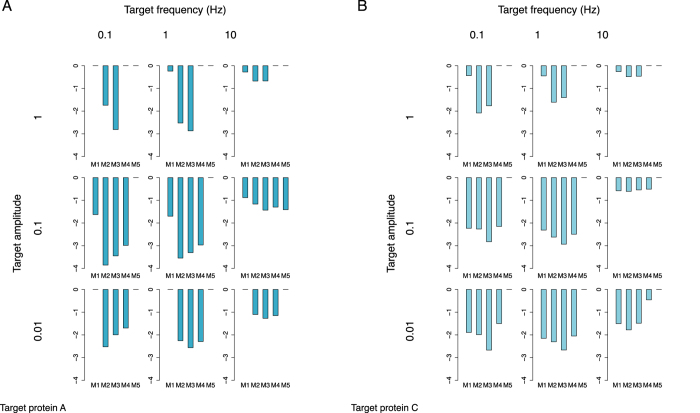
Unordered score profiles containing the logarithm of the HVUPF values obtained for each model structure under different experimental conditions (target frequency (Hz) and amplitude (conc.)) for observed active concentrations of (**A**) protein A and (**B**) protein C (a lower value is better). Each of the nine bar plots (score profiles) shows the obtained HVUPF (y-axis of the bar plots) of each model structure for the specific pair of target frequency and amplitude. The model enumeration (x-axis of the bar plots) is the same as in Fig. 5C. The obtained median ranks for (M1, M2, M3, M4, M5) are (4, 2, 1, 3, 4.5) and (3, 1, 2, 4, 5) for the observed target active concentrations of proteins A and C, respectively.

Figure 6 shows the HVUPF for each model for each design target. All of the models have the same number of reactions (considering M1 to contain an auto-interaction with *k* = 0). Therefore, the complexity component of the function used for scoring (equation (2)) is the same for all candidate models. Subsequently, we ranked the models only by their HVUPF. From both Fig. 6A,B, it is evident that the candidate models M2 and M3 consistently dominated the other models in all experimental setups, confirming the conclusions from the study by Tsai *et al*.^18^ regarding the wide operational range (tunable frequency and constancy of amplitude), i.e. the robustness of the model structures containing an auto-activation loop and further confirming the validity of our approach. For the model structures M2 and M3, the frequency operational range is extended towards the lower frequencies and the amplitude constancy range towards the higher end. The influence of the auto-activation loop is more expressed in the experiments where the target is the active concentration of protein A. The model structures containing auto-inhibition (M4, M5) do not offer significant improvement and extension of the operational range over the basic model structure M1.

The rankings for target frequency 1 Hz and amplitude 0.1 correspond to the rankings obtained in the study by Barnes *et al*.^8^ for the target expected properties of the behaviours of both proteins A and C. The difference in the rankings in both cases is the ranking of model structure M1 (negative feedback without auto-interaction) and model structure M4 (negative feedback with weak auto-inhibition). In our results, for this specific pair of target frequency and amplitude, M4 performs better than M1. Note that the two-dimensional projections of the Pareto front and the simulations for the best performing model structures M2 and M3, obtained using target amplitude *A*_*t*_ = 0.1 and different target frequencies, are given in the Supplementary. Supplementary Figures S3–S5 show those obtained for scenarios with observed target active concentrations of protein A and Supplementary Figs S6–S8 show those obtained for scenarios with observed target active concentrations of protein C.

## Discussion

The paper introduces process-based design, a novel approach to designing dynamical biological systems that exhibit desired behaviours. The process-based formalism we use allows for flexible and modular representation of modelling knowledge in the domain of interest, formalized as a taxonomy of template entities (constituents) and processes (interactions). We present ProBMoTd, an automated design tool that can make use of such knowledge by instantiating the templates into reusable components for building candidate models, which are put together into model structures in a manner similar to the one used by composition-based approaches^5^,^6^. It automatically resolves the structural uncertainty by enumerating and exploring the space of candidate models. Furthermore, ProBMoTd resolves the parametric uncertainty by using standard multi-objective optimization methods, as the ones used by optimization-based approaches^8^,^11^,^13^. It fits the values of the model parameters to the expected properties of the desired behaviour, obtaining a Pareto front on non-dominated optimal solutions. Finally, ProBMoTd combines the hyper-volume under the Pareto front with the complexity of the model structure to obtain a single score used for ranking the candidate designs. The process-based design is closely related to TinkerCell^28^, which employs a hierarchical representation of domain knowledge, but, in contrast to our approach, limits its focus to manual resolution of the structural and parametric uncertainties of the design task.

We illustrate the utility and generality of the process-based design approach on two design tasks. In the first, we design a stochastic toggle switch without cooperativity, while in the second, we design a deterministic oscillator. The experiments show that our approach is general enough to handle design tasks based on either deterministic or stochastic models. Furthermore, in both experiments, the ranking of candidate models based on the hyper-volume under the Pareto front of optimal solutions resembles the rankings reported in previous studies. This shows the utility of the hyper-volume measure as a design-selection strategy: It successfully summarizes the set of optimal solutions, produced by optimizing multiple competing objectives, into a single ranking score. In contrast to existing optimization-based approaches, process-based design facilitates modular knowledge representation, allowing for flexible specification of arbitrarily complex spaces of candidate model structures. This allowed us to easily specify a superset of the space of candidate model structures considered in previous studies, which subsequently led us to the discovery of previously unconsidered candidate designs of a stochastic toggle switch without cooperativity. Subsequently, we gained additional knowledge about the influence of the different choices of component processes on the overall model performance. Finally, when designing a robust oscillator, due to the automation of the complete ProBMoT workflow, we were able to efficiently perform a range of experiments with different setups, the results of which improved the confidence in the generality and robustness of the designs reported in previous studies.

The work presented in this paper lays the foundation for process-based design. The further development of the proposed approach depends on resolving its current limitations. Most importantly, the process-based design approach has limited scalability to large spaces of candidate designs. This is due to the fact that structural uncertainty is currently resolved by exhaustive enumeration of constrained spaces of candidate designs. This limitation can be overcome by replacing exhaustive enumeration with incomplete search guided by heuristics^29^. In particular, one direction is the development of a representation for the structural uncertainty within an incomplete process-based model at the level of parametric uncertainty. In this way, the process-based design approach will come closer to the optimization-based approaches. An additional direction to be explored is the definition of efficient structural refinement operators that can be used by standard search methods for exploring the space of candidates. Another limitation of the process-based design approach that we would like to mention is its current non-interoperability with synthetic biology standards. Currently, process-based models produced by ProBMoTd can be recoded only into the Systems Biology Markup Language^30^. A large step towards interoperability will be the construction and annotation of libraries of domain knowledge by transferring knowledge from registries of standard parts^31^ or similar standardization efforts^32^. This will allow for creating designs partly or completely composed of readily available biological components. It will also allow for translation of process-based models into standard formats specific to synthetic biology, such as the Synthetic Biology Open Language^33^. Addressing the above limitations will improve the efficiency of the process-based design task, as well as the understandability and communicability of the proposed designs, thus facilitating the further application to problems coming from the domain of synthetic biology.

## Additional Information

**How to cite this article**: Tanevski, J. *et al*. Process-based design of dynamical biological systems. *Sci. Rep.* **6**, 34107; doi: 10.1038/srep34107 (2016).

## Supplementary Material

Supplementary Information
