# Supplementary material for: Process-based design of dynamical biological systems
Source: Sci Rep. 2016 Sep 30;6:34107. doi: 10.1038/srep34107 (PMC5043446; doi:10.1038/srep34107)
Supplement: Supplementary Information [file srep34107-s1.pdf]

Process-based design of dynamical biological systems –Supplementary material–

Jovan Tanevski, Ljupčo Todorovski and Sašo Džeroski

Contents

1 Availability of software **2**

2 Stochastic toggle switch without cooperativity **2**
2.1 Formal representation of the library and incomplete model 2
2.2 Candidate model equations 8
2.3 Supplementary figures 15

3 Robust negative feedback oscillations **16**
3.1 Formal representation of the library and incomplete model 16
3.2 Candidate model equations 17
3.3 Supplementary figures 20

1 Availability of software

The ProBMoTd tool is an extension of ProBMoT that addresses the task of process-based design as described in the manuscript. Both ProBMoTd and ProBMoT are released as open-source software packages available for download at http://probmot.ijs.si. They are available under the terms of the three-clause BSD license (http://probmot.ijs.si/licence.html)

2 Stochastic toggle switch without cooperativity

2.1 Formal representation of the library and incomplete model

The complete library used for the task of design of a stochastic toggle switch without cooperativity (graphically depicted in Fig. 2 in the manuscript) is shown in Table S1. The incomplete model used to enumerate the 20 candidate models considered in this task is shown in Table S2.

Table S1: Formal representation of the library of domain knowledge used for the design of a stochastic toggle switch without cooperativity.

```
library ToggleLibrary;

template entity gene{
  vars: mol {range:<0,100>};
}

template entity protein{
  vars: mol {range:<0,100>};
  consts:
    trate {range: <0.001,50>},
    drate {range: <0.001,5>}
}

template entity complex{
  vars: mol {range:<0,100>};
}

template entity inducer{
  vars: mol {range:<0,100>};
}

template entity bound_factor{
  vars: mol {range:<0,100>};
}
```

continued . . .

Table S1: Formal representation of the library of domain knowledge used for the design of a stochastic toggle switch without cooperativity.

```
template process basic(g: gene, p: protein){
  processes:
    production(g, p),
    degradation(p);
}

template process production(g: gene, p: protein){
  equations:
    g.mol -> g.mol + p.mol [p.trate];
}

template process degradation(p: protein){
  equations:
    p.mol ->  [p.drate];
}

template process reversible_binding(p1: protein, g2: gene,
                  p2:protein, g1: gene, p1g2: bound_factor,
                            p2g1: bound_factor, e: protein){
  consts:
    k1{range: <0.001, 50>},
    k2{range: <0.001, 50>},
    k3{range: <0.001, 50>},
    k4{range: <0.001, 50>};
}

template process reversible_binding_simple : reversible_binding{
  processes:
    single_reversible_binding(p1, g2, p1g2),
    single_reversible_binding(p2, g1, p2g1),
    bound_decay(p1g2, g2, p2g1, g1);
}

template process reversible_binding_exclusive
                                        : reversible_binding{
  processes:
    bound_decay_e(p1g2, g2, p2g1, g1, e);
  equations:
    p1.mol + g2.mol + e.mol ->  p1g2.mol [k1],
    p1g2.mol -> p1.mol + g2.mol + e.mol [k2],
    p2.mol + g1.mol + e.mol ->  p2g1.mol [k3],
    p2g1.mol -> p2.mol + g1.mol + e.mol [k4];
}
```

continued ...

Table S1: Formal representation of the library of domain knowledge used for the design of a stochastic toggle switch without cooperativity.

```
template process single_reversible_binding(p: protein, g: gene,
                                               pg: bound_factor){
  consts:
    k1{range: <0.001, 50>},
    k2{range: <0.001, 50>};
}

template process single_reversible_binding_none
                                   : single_reversible_binding{}

template process single_reversible_binding_simple
                                   : single_reversible_binding{
  equations:
    p.mol + g.mol ->  pg.mol [k1],
    pg.mol -> p.mol + g.mol [k2];
}

template process bound_decay(p1g2: bound_factor, g2: gene,
                                p2g1: bound_factor, g1: gene){
  consts:
    k1{range: <0.001, 50>},
    k2{range: <0.001, 50>};
}

template process bound_decay_none : bound_decay{}

template process bound_decay_present : bound_decay{
  equations:
    p1g2.mol -> g2.mol [k1],
    p2g1.mol -> g1.mol [k2];
}

template process bound_decay_e(p1g2: bound_factor, g2: gene,
                   p2g1: bound_factor, g1: gene, e: protein){
  consts:
    k1{range: <0.001, 50>},
    k2{range: <0.001, 50>};
}
```

continued . . .

Table S1: Formal representation of the library of domain knowledge used for the design of a stochastic toggle switch without cooperativity.

```
template process bound_decay_e_none : bound_decay_e{}

template process bound_decay_e_present : bound_decay_e{
  equations:
    p1g2.mol -> g2.mol + e.mol [k1],
    p2g1.mol -> g1.mol + e.mol [k2];
}

template process complex_formation(i: inducer, p: protein,
                                               ip: complex){
  consts:
    k{range: <0.001, 50>};
  equations:
    i.mol + p.mol -> ip.mol [k];
}

template process protein_binding(p1: protein, p2: protein,
                                              p1p2: complex){
  consts:
    k1{range: <0.001, 50>},
    k2{range: <0.001, 50>};
}

template process protein_binding_none : protein_binding {}

template process protein_binding_present : protein_binding{
  equations:
    p1.mol + p2.mol -> p1p2.mol [k1],
    p1p2.mol -> p1.mol + p2.mol [k2];
}
```

Table S2: Formal representation of the incomplete model used for the design of a stochastic toggle switch without cooperativity.

```
incomplete model ToggleSwitch : ToggleLibrary;

entity gA : gene{
  vars: mol{role: endogenous; initial: 1;};
}

entity gB : gene{
  vars: mol{role: endogenous; initial: 1;};
}

entity A : protein{
  vars: mol{role: endogenous; initial: 0;};
  consts: trate, drate;
}

entity B : protein{
  vars: mol{role: endogenous; initial: 0;};
  consts: trate, drate;
}

entity P : protein{
  vars: mol{role: endogenous; initial: 1;};
  consts: trate, drate;
}

entity SA : complex{
  vars: mol{role: endogenous; initial: 0;};
}

entity RB : complex{
  vars: mol{role: endogenous; initial: 0;};
}

entity AB : complex{
  vars: mol{role: endogenous; initial: 0;};
}

entity S : inducer{
  vars: mol{role: exogenous; initial: 0};
}
```

continued ...

Table S2: Formal representation of the incomplete model used for the design of a stochastic toggle switch without cooperativity.

```
entity R : inducer{
  vars: mol{role: exogenous; initial: 0};
}

entity AgB : bound_factor{
  vars: mol{role: endogenous; initial: 0;};
}

entity BgA : bound_factor{
  vars: mol{role: endogenous; initial: 0;};
}

process basicA(gA, A) : basic {}
process basicB(gB, B) : basic {}

process complex_formationSA(S, A, SA) : complex_formation{
  consts: k;
}

process complex_formationRB(R,B,RB) : complex_formation{
  consts: k;
}

process reversible_bindingAgBBgA(A, gB, B, gA, AgB, BgA, P)
                                   : reversible_binding{
  consts: k1, k2, k3, k4;
}

process protein_bindingAB(A, B, AB) : protein_binding {
  consts: k1, k2;
}
```

2.2 Candidate model equations

Below we give the complete descriptions of the reactions and the corresponding rates for all candidate models considered for the task of designing a stochastic toggle switch without cooperativity.

Model 1: HVUPF=0.712234502, Complexity=6, Rank=15

Reaction	Rate
$A.mol \rightarrow$	$A.drate$
$B.mol \rightarrow$	$B.drate$
$gA.mol \rightarrow gA.mol + A.mol$	$A.trate$
$gB.mol \rightarrow gB.mol + B.mol$	$B.trate$
$R.mol + B.mol \rightarrow RB.mol$	$complex\_formationRB.k$
$S.mol + A.mol \rightarrow SA.mol$	$complex\_formationSA.k$

Model 2: HVUPF=0.426312245, Complexity=8, Rank=6

Reaction	Rate
$A.mol + gB.mol \rightarrow AgB.mol$	$single\_reversible\_binding.k1$
$A.mol \rightarrow$	$A.drate$
$AgB.mol \rightarrow A.mol + gB.mol$	$single\_reversible\_binding.k2$
$B.mol \rightarrow$	$B.drate$
$gA.mol \rightarrow gA.mol + A.mol$	$A.trate$
$gB.mol \rightarrow gB.mol + B.mol$	$B.trate$
$R.mol + B.mol \rightarrow RB.mol$	$complex\_formationRB.k$
$S.mol + A.mol \rightarrow SA.mol$	$complex\_formationSA.k$

Model 3: HVUPF=0.698638372, Complexity=8, Rank=17

Reaction	Rate
$A.mol \rightarrow$	$A.drate$
$B.mol + gA.mol \rightarrow BgA.mol$	$single\_reversible\_binding.k1$
$B.mol \rightarrow$	$B.drate$
$BgA.mol \rightarrow B.mol + gA.mol$	$single\_reversible\_binding.k2$
$gA.mol \rightarrow gA.mol + A.mol$	$A.trate$
$gB.mol \rightarrow gB.mol + B.mol$	$B.trate$
$R.mol + B.mol \rightarrow RB.mol$	$complex\_formationRB.k$
$S.mol + A.mol \rightarrow SA.mol$	$complex\_formationSA.k$

Model 4: HVUPF=0.457644189, Complexity=10, Rank=13

Reaction	Rate
$A.mol + gB.mol \to AgB.mol$	$single\_reversible\_binding\_AgB.k1$
$A.mol \to$	$A.drate$
$AgB.mol \to A.mol + gB.mol$	$single\_reversible\_binding\_AgB.k2$
$B.mol + gA.mol \to BgA.mol$	$single\_reversible\_binding\_BgA.k1$
$B.mol \to$	$B.drate$
$BgA.mol \to B.mol + gA.mol$	$single\_reversible\_binding\_BgA.k2$
$gA.mol \to gA.mol + A.mol$	$A.trate$
$gB.mol \to gB.mol + B.mol$	$B.trate$
$R.mol + B.mol \to RB.mol$	$complex\_formationRB.k$
$S.mol + A.mol \to SA.mol$	$complex\_formationSA.k$

Model 5: HVUPF=0.837428418, Complexity=8, Rank=19

Reaction	Rate
$A.mol \to$	$A.drate$
$AgB.mol \to gB.mol$	$bound\_decay.k1$
$B.mol \to$	$B.drate$
$BgA.mol \to gA.mol$	$bound\_decay.k2$
$gA.mol \to gA.mol + A.mol$	$A.trate$
$gB.mol \to gB.mol + B.mol$	$B.trate$
$R.mol + B.mol \to RB.mol$	$complex\_formationRB.k$
$S.mol + A.mol \to SA.mol$	$complex\_formationSA.k$

Model 6: HVUPF=0.392625279, Complexity=10, Rank=11

Reaction	Rate
$A.mol + gB.mol \to AgB.mol$	$single\_reversible\_binding.k1$
$A.mol \to$	$A.drate$
$AgB.mol \to A.mol + gB.mol$	$single\_reversible\_binding.k2$
$AgB.mol \to gB.mol$	$bound\_decay.k1$
$B.mol \to$	$B.drate$
$BgA.mol \to gA.mol$	$bound\_decay.k2$
$gA.mol \to gA.mol + A.mol$	$A.trate$
$gB.mol \to gB.mol + B.mol$	$B.trate$
$R.mol + B.mol \to RB.mol$	$complex\_formationRB.k$
$S.mol + A.mol \to SA.mol$	$complex\_formationSA.k$

Model 7: HVUPF=0.810606879, Complexity=10, Rank=20

Reaction	Rate
$A.mol \rightarrow$	$A.drate$
$AgB.mol \rightarrow gB.mol$	$bound\_decay.k1$
$B.mol + gA.mol \rightarrow BgA.mol$	$single\_reversible\_binding.k1$
$B.mol \rightarrow$	$B.drate$
$BgA.mol \rightarrow B.mol + gA.mol$	$single\_reversible\_binding.k2$
$BgA.mol \rightarrow gA.mol$	$bound\_decay.k2$
$gA.mol \rightarrow gA.mol + A.mol$	$A.trate$
$gB.mol \rightarrow gB.mol + B.mol$	$B.trate$
$R.mol + B.mol \rightarrow RB.mol$	$complex\_formationRB.k$
$S.mol + A.mol \rightarrow SA.mol$	$complex\_formationSA.k$

Model 8: HVUPF=0.294097735, Complexity=12, Rank=12

Reaction	Rate
$A.mol + gB.mol \rightarrow AgB.mol$	$single\_reversible\_binding\_AgB.k1$
$A.mol \rightarrow$	$A.drate$
$AgB.mol \rightarrow A.mol + gB.mol$	$single\_reversible\_binding\_AgB.k2$
$AgB.mol \rightarrow gB.mol$	$bound\_decay.k1$
$B.mol + gA.mol \rightarrow BgA.mol$	$single\_reversible\_binding\_BgA.k1$
$B.mol \rightarrow$	$B.drate$
$BgA.mol \rightarrow B.mol + gA.mol$	$single\_reversible\_binding\_BgA.k2$
$BgA.mol \rightarrow gA.mol$	$bound\_decay.k2$
$gA.mol \rightarrow gA.mol + A.mol$	$A.trate$
$gB.mol \rightarrow gB.mol + B.mol$	$B.trate$
$R.mol + B.mol \rightarrow RB.mol$	$complex\_formationRB.k$
$S.mol + A.mol \rightarrow SA.mol$	$complex\_formationSA.k$

Model 9: HVUPF=0.173166305, Complexity=10, Rank=1

Reaction	Rate
$A.mol + gB.mol + P.mol \rightarrow AgB.mol$	$reversible\_bindingAgBBgA.k1$
$A.mol \rightarrow$	$A.drate$
$AgB.mol \rightarrow A.mol + gB.mol + P.mol$	$reversible\_bindingAgBBgA.k2$
$B.mol + gA.mol + P.mol \rightarrow BgA.mol$	$reversible\_bindingAgBBgA.k3$
$B.mol \rightarrow$	$B.drate$
$BgA.mol \rightarrow B.mol + gA.mol + P.mol$	$reversible\_bindingAgBBgA.k4$
$gA.mol \rightarrow gA.mol + A.mol$	$A.trate$
$gB.mol \rightarrow gB.mol + B.mol$	$B.trate$
$R.mol + B.mol \rightarrow RB.mol$	$complex\_formationRB.k$
$S.mol + A.mol \rightarrow SA.mol$	$complex\_formationSA.k$

Model 10: HVUPF=0.159207006, Complexity=12, Rank=4

Reaction	Rate
$A.mol + gB.mol + P.mol \rightarrow AgB.mol$	$reversible\_bindingAgBBgA.k1$
$A.mol \rightarrow$	$A.drate$
$AgB.mol \rightarrow A.mol + gB.mol + P.mol$	$reversible\_bindingAgBBgA.k2$
$AgB.mol \rightarrow gB.mol + P.mol$	$bound\_decay\_e.k1$
$B.mol + gA.mol + P.mol \rightarrow BgA.mol$	$reversible\_bindingAgBBgA.k3$
$B.mol \rightarrow$	$B.drate$
$BgA.mol \rightarrow B.mol + gA.mol + P.mol$	$reversible\_bindingAgBBgA.k4$
$BgA.mol \rightarrow gA.mol + P.mol$	$bound\_decay\_e.k2$
$gA.mol \rightarrow gA.mol + A.mol$	$A.trate$
$gB.mol \rightarrow gB.mol + B.mol$	$B.trate$
$R.mol + B.mol \rightarrow RB.mol$	$complex\_formationRB.k$
$S.mol + A.mol \rightarrow SA.mol$	$complex\_formationSA.k$

Model 11: HVUPF=0.414773371, Complexity=8, Rank=5

Reaction	Rate
$A.mol + B.mol \rightarrow AB.mol$	$protein\_bindingAB.k1$
$A.mol \rightarrow$	$A.drate$
$AB.mol \rightarrow A.mol + B.mol$	$protein\_bindingAB.k2$
$B.mol \rightarrow$	$B.drate$
$gA.mol \rightarrow gA.mol + A.mol$	$A.trate$
$gB.mol \rightarrow gB.mol + B.mol$	$B.trate$
$R.mol + B.mol \rightarrow RB.mol$	$complex\_formationRB.k$
$S.mol + A.mol \rightarrow SA.mol$	$complex\_formationSA.k$

Model 12: HVUPF=0.331451333, Complexity=10, Rank=7

Reaction	Rate
$A.mol + B.mol \rightarrow AB.mol$	$protein\_bindingAB.k1$
$A.mol + gB.mol \rightarrow AgB.mol$	$single\_reversible\_binding.k1$
$A.mol \rightarrow$	$A.drate$
$AB.mol \rightarrow A.mol + B.mol$	$protein\_bindingAB.k2$
$AgB.mol \rightarrow A.mol + gB.mol$	$single\_reversible\_binding.k2$
$B.mol \rightarrow$	$B.drate$
$gA.mol \rightarrow gA.mol + A.mol$	$A.trate$
$gB.mol \rightarrow gB.mol + B.mol$	$B.trate$
$R.mol + B.mol \rightarrow RB.mol$	$complex\_formationRB.k$
$S.mol + A.mol \rightarrow SA.mol$	$complex\_formationSA.k$

Model 13: HVUPF=0.363104873, Complexity=10, Rank=9

Reaction	Rate
$A.mol + B.mol \rightarrow AB.mol$	$protein\_bindingAB.k1$
$A.mol \rightarrow$	$A.drate$
$AB.mol \rightarrow A.mol + B.mol$	$protein\_bindingAB.k2$
$B.mol + gA.mol \rightarrow BgA.mol$	$single\_reversible\_binding.k1$
$B.mol \rightarrow$	$B.drate$
$BgA.mol \rightarrow B.mol + gA.mol$	$single\_reversible\_binding.k2$
$gA.mol \rightarrow gA.mol + A.mol$	$A.trate$
$gB.mol \rightarrow gB.mol + B.mol$	$B.trate$
$R.mol + B.mol \rightarrow RB.mol$	$complex\_formationRB.k$
$S.mol + A.mol \rightarrow SA.mol$	$complex\_formationSA.k$

Model 14: HVUPF=0.153544691, Complexity=12, Rank=3

Reaction	Rate
$A.mol + B.mol \rightarrow AB.mol$	$protein\_bindingAB.k1$
$A.mol + gB.mol \rightarrow AgB.mol$	$single\_reversible\_binding\_AgB.k1$
$A.mol \rightarrow$	$A.drate$
$AB.mol \rightarrow A.mol + B.mol$	$protein\_bindingAB.k2$
$AgB.mol \rightarrow A.mol + gB.mol$	$single\_reversible\_binding\_AgB.k2$
$B.mol + gA.mol \rightarrow BgA.mol$	$single\_reversible\_binding\_BgA.k1$
$B.mol \rightarrow$	$B.drate$
$BgA.mol \rightarrow B.mol + gA.mol$	$single\_reversible\_binding\_BgA.k2$
$gA.mol \rightarrow gA.mol + A.mol$	$A.trate$
$gB.mol \rightarrow gB.mol + B.mol$	$B.trate$
$R.mol + B.mol \rightarrow RB.mol$	$complex\_formationRB.k$
$S.mol + A.mol \rightarrow SA.mol$	$complex\_formationSA.k$

Model 15: HVUPF=0.384784585, Complexity=10, Rank=10

Reaction	Rate
$A.mol + B.mol \rightarrow AB.mol$	$protein\_bindingAB.k1$
$A.mol \rightarrow$	$A.drate$
$AB.mol \rightarrow A.mol + B.mol$	$protein\_bindingAB.k2$
$AgB.mol \rightarrow gB.mol$	$bound\_decay.k1$
$B.mol \rightarrow$	$B.drate$
$BgA.mol \rightarrow gA.mol$	$bound\_decay.k2$
$gA.mol \rightarrow gA.mol + A.mol$	$A.trate$
$gB.mol \rightarrow gB.mol + B.mol$	$B.trate$
$R.mol + B.mol \rightarrow RB.mol$	$complex\_formationRB.k$
$S.mol + A.mol \rightarrow SA.mol$	$complex\_formationSA.k$

Model 16: HVUPF=0.339729811, Complexity=12, Rank=14

Reaction	Rate
$A.mol + B.mol \to AB.mol$	$protein\_bindingAB.k1$
$A.mol + gB.mol \to AgB.mol$	$single\_reversible\_binding.k1$
$A.mol \to$	$A.drate$
$AB.mol \to A.mol + B.mol$	$protein\_bindingAB.k2$
$AgB.mol \to A.mol + gB.mol$	$single\_reversible\_binding.k2$
$AgB.mol \to gB.mol$	$bound\_decay.k1$
$B.mol \to$	$B.drate$
$BgA.mol \to gA.mol$	$bound\_decay.k2$
$gA.mol \to gA.mol + A.mol$	$A.trate$
$gB.mol \to gB.mol + B.mol$	$B.trate$
$R.mol + B.mol \to RB.mol$	$complex\_formationRB.k$
$S.mol + A.mol \to SA.mol$	$complex\_formationSA.k$

Model 17: HVUPF=0.40783702, Complexity=12, Rank=16

Reaction	Rate
$A.mol + B.mol \to AB.mol$	$protein\_bindingAB.k1$
$A.mol \to$	$A.drate$
$AB.mol \to A.mol + B.mol$	$protein\_bindingAB.k2$
$AgB.mol \to gB.mol$	$bound\_decay.k1$
$B.mol + gA.mol \to BgA.mol$	$single\_reversible\_binding.k1$
$B.mol \to$	$B.drate$
$BgA.mol \to B.mol + gA.mol$	$single\_reversible\_binding.k2$
$BgA.mol \to gA.mol$	$bound\_decay.k2$
$gA.mol \to gA.mol + A.mol$	$A.trate$
$gB.mol \to gB.mol + B.mol$	$B.trate$
$R.mol + B.mol \to RB.mol$	$complex\_formationRB.k$
$S.mol + A.mol \to SA.mol$	$complex\_formationSA.k$

Model 18: HVUPF=0.347634959, Complexity=14, Rank=18

Reaction	Rate
$A.mol + B.mol \to AB.mol$	$protein\_bindingAB.k1$
$A.mol + gB.mol \to AgB.mol$	$single\_reversible\_binding\_AgB.k1$
$A.mol \to$	$A.drate$
$AB.mol \to A.mol + B.mol$	$protein\_bindingAB.k2$
$AgB.mol \to A.mol + gB.mol$	$single\_reversible\_binding\_AgB.k2$
$AgB.mol \to gB.mol$	$bound\_decay.k1$
$B.mol + gA.mol \to BgA.mol$	$single\_reversible\_binding\_BgA.k1$
$B.mol \to$	$B.drate$
$BgA.mol \to B.mol + gA.mol$	$single\_reversible\_binding\_BgA.k2$
$BgA.mol \to gA.mol$	$bound\_decay.k2$
$gA.mol \to gA.mol + A.mol$	$A.trate$
$gB.mol \to gB.mol + B.mol$	$B.trate$
$R.mol + B.mol \to RB.mol$	$complex\_formationRB.k$
$S.mol + A.mol \to SA.mol$	$complex\_formationSA.k$

Model 19: HVUPF=0.093538518, Complexity=12, Rank=2

Reaction	Rate
$A.mol + B.mol \rightarrow AB.mol$	$protein\_bindingAB.k1$
$A.mol + gB.mol + P.mol \rightarrow AgB.mol$	$reversible\_bindingAgBBgA.k1$
$A.mol \rightarrow$	$A.drate$
$AB.mol \rightarrow A.mol + B.mol$	$protein\_bindingAB.k2$
$AgB.mol \rightarrow A.mol + gB.mol + P.mol$	$reversible\_bindingAgBBgA.k2$
$B.mol + gA.mol + P.mol \rightarrow BgA.mol$	$reversible\_bindingAgBBgA.k3$
$B.mol \rightarrow$	$B.drate$
$BgA.mol \rightarrow B.mol + gA.mol + P.mol$	$reversible\_bindingAgBBgA.k4$
$gA.mol \rightarrow gA.mol + A.mol$	$A.trate$
$gB.mol \rightarrow gB.mol + B.mol$	$B.trate$
$R.mol + B.mol \rightarrow RB.mol$	$complex\_formationRB.k$
$S.mol + A.mol \rightarrow SA.mol$	$complex\_formationSA.k$

Model 20: HVUPF=0.106389079, Complexity=14, Rank=8

Reaction	Rate
$A.mol + B.mol \rightarrow AB.mol$	$protein\_bindingAB.k1$
$A.mol + gB.mol + P.mol \rightarrow AgB.mol$	$reversible\_bindingAgBBgA.k1$
$A.mol \rightarrow$	$A.drate$
$AB.mol \rightarrow A.mol + B.mol$	$protein\_bindingAB.k2$
$AgB.mol \rightarrow A.mol + gB.mol + P.mol$	$reversible\_bindingAgBBgA.k2$
$AgB.mol \rightarrow gB.mol + P.mol$	$bound\_decay\_e.k1$
$B.mol + gA.mol + P.mol \rightarrow BgA.mol$	$reversible\_bindingAgBBgA.k3$
$B.mol \rightarrow$	$B.drate$
$BgA.mol \rightarrow B.mol + gA.mol + P.mol$	$reversible\_bindingAgBBgA.k4$
$BgA.mol \rightarrow gA.mol + P.mol$	$bound\_decay\_e.k2$
$gA.mol \rightarrow gA.mol + A.mol$	$A.trate$
$gB.mol \rightarrow gB.mol + B.mol$	$B.trate$
$R.mol + B.mol \rightarrow RB.mol$	$complex\_formationRB.k$
$S.mol + A.mol \rightarrow SA.mol$	$complex\_formationSA.k$

2.3 Supplementary figures

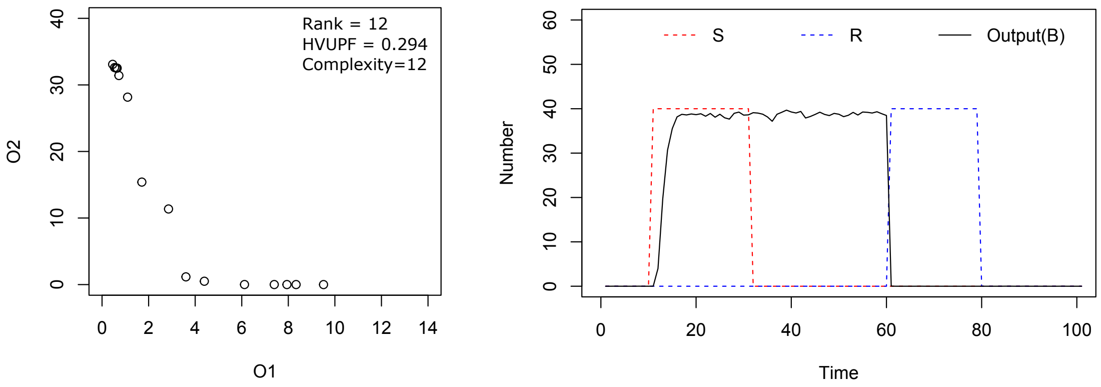

Figure S1: (left) Pareto front for the model structure with added bound degradation only (ranked 12th). (right) Simulation of the observed output variable (average of 100 realizations) using the model structure and an arbitrarily selected set of optimal parameter values from the Pareto front (single point).

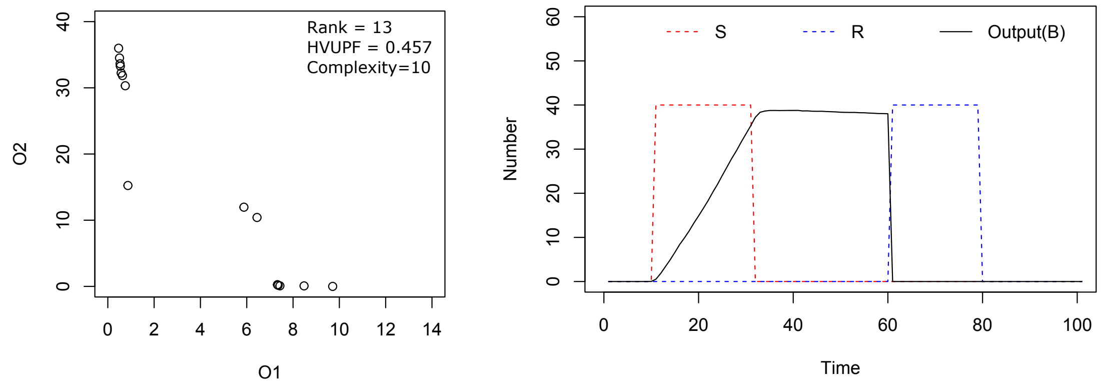

Figure S2: (left) Pareto front for the model structure describing a simple double repressed toggle switch without cooperativity (ranked 13th). (right) Simulation of the observed output variable (average of 100 realizations) using the model structure and an arbitrarily selected set of optimal parameter values from the Pareto front (single point).

3 Robust negative feedback oscillations

3.1 Formal representation of the library and incomplete model

The complete library is shown on Figure 4A in the manuscript. The incomplete model is shown in Table S3.

Table S3: Formal representation of the incomplete model used for the design of a robust negative feedback oscillatior.

```
incomplete model RobustOscillator : OscillatorNetworks;

entity A : Protein{
  vars:
    aconc{role: endogenous; initial: 0},
    iconc{role: endogenous; initial: 1};
}
entity B : Protein{
  vars:
    aconc{role: endogenous; initial: 0},
    iconc{role: endogenous; initial: 1};
}
entity C : Protein{
  vars:
    aconc{role: endogenous; initial: 0},
    iconc{role: endogenous; initial: 1};
}

process linkAB(A, B) : Inhibition { consts: k, K, n; }
process linkBC(B, C) : Inhibition { consts: k, K, n; }
process linkCA(C, A) : Inhibition { consts: k, K, n; }

process linkAA(A) : AutoInteraction {
  consts: kw, ks, K, n;
}

process AConv(A) : Conversion { consts: k; }
process BConv(B) : Conversion { consts: k; }
process CConv(C) : Conversion { consts: k=1; }
```

3.2 Candidate model equations

Model1

$$
\begin{aligned}
\frac{dA.aconc}{dt} &= k_1 A.iconc - \frac{k_2 C.aconc^{n_1}}{K_1^{n_1} + C.aconc^{n_1}} A.aconc \\
\frac{dA.iconc}{dt} &= -k_1 A.iconc + \frac{k_2 C.aconc^{n_1}}{K_1^{n_1} + C.aconc^{n_1}} A.aconc \\
\frac{dB.aconc}{dt} &= k_3 B.iconc - \frac{k_4 A.aconc^{n_2}}{K_2^{n_2} + A.aconc^{n_2}} B.aconc \\
\frac{dB.iconc}{dt} &= -k_3 B.iconc + \frac{k_4 A.aconc^{n_2}}{K_2^{n_2} + A.aconc^{n_2}} B.aconc \\
\frac{dC.aconc}{dt} &= k_5 C.iconc - \frac{k_6 B.aconc^{n_3}}{K_3^{n_3} + B.aconc^{n_3}} C.aconc \\
\frac{dC.iconc}{dt} &= -k_5 C.iconc + \frac{k_6 B.aconc^{n_3}}{K_3^{n_3} + B.aconc^{n_3}} C.aconc
\end{aligned}
$$

Model 2

$$
\begin{aligned}
\frac{dA.aconc}{dt} &= k_1 A.iconc - \frac{k_2 C.aconc^{n_1}}{K_1^{n_1} + C.aconc^{n_1}} A.aconc + k_w A.iconc \frac{A.aconc^{n_4}}{K_4^{n_4} + A.aconc^{n_4}} \\
\frac{dA.iconc}{dt} &= -k_1 A.iconc + \frac{k_2 C.aconc^{n_1}}{K_1^{n_1} + C.aconc^{n_1}} A.aconc - k_w A.iconc \frac{A.aconc^{n_4}}{K_4^{n_4} + A.aconc^{n_4}} \\
\frac{dB.aconc}{dt} &= k_3 B.iconc - \frac{k_4 A.aconc^{n_2}}{K_2^{n_2} + A.aconc^{n_2}} B.aconc \\
\frac{dB.iconc}{dt} &= -k_3 B.iconc + \frac{k_4 A.aconc^{n_2}}{K_2^{n_2} + A.aconc^{n_2}} B.aconc \\
\frac{dC.aconc}{dt} &= k_5 C.iconc - \frac{k_6 B.aconc^{n_3}}{K_3^{n_3} + B.aconc^{n_3}} C.aconc \\
\frac{dC.iconc}{dt} &= -k_5 C.iconc + \frac{k_6 B.aconc^{n_3}}{K_3^{n_3} + B.aconc^{n_3}} C.aconc
\end{aligned}
$$

Model 3

$$\begin{aligned}
\frac{dA.aconc}{dt} &= k_1 A.iconc - \frac{k_2 C.aconc^{n_1}}{K_1^{n_1} + C.aconc^{n_1}} A.aconc + k_s A.iconc \frac{A.aconc^{n_4}}{K_4^{n_4} + A.aconc^{n_4}} \\
\frac{dA.iconc}{dt} &= -k_1 A.iconc + \frac{k_2 C.aconc^{n_1}}{K_1^{n_1} + C.aconc^{n_1}} A.aconc - k_s A.iconc \frac{A.aconc^{n_4}}{K_4^{n_4} + A.aconc^{n_4}} \\
\frac{dB.aconc}{dt} &= k_3 B.iconc - \frac{k_4 A.aconc^{n_2}}{K_2^{n_2} + A.aconc^{n_2}} B.aconc \\
\frac{dB.iconc}{dt} &= -k_3 B.iconc + \frac{k_4 A.aconc^{n_2}}{K_2^{n_2} + A.aconc^{n_2}} B.aconc \\
\frac{dC.aconc}{dt} &= k_5 C.iconc - \frac{k_6 B.aconc^{n_3}}{K_3^{n_3} + B.aconc^{n_3}} C.aconc \\
\frac{dC.iconc}{dt} &= -k_5 C.iconc + \frac{k_6 B.aconc^{n_3}}{K_3^{n_3} + B.aconc^{n_3}} C.aconc
\end{aligned}$$

Model 4

$$\begin{aligned}
\frac{dA.aconc}{dt} &= k_1 A.iconc - \frac{k_2 C.aconc^{n_1}}{K_1^{n_1} + C.aconc^{n_1}} A.aconc - k_w A.aconc \frac{A.aconc^{n_4}}{K_4^{n_4} + A.aconc^{n_4}} \\
\frac{dA.iconc}{dt} &= -k_1 A.iconc + \frac{k_2 C.aconc^{n_1}}{K_1^{n_1} + C.aconc^{n_1}} A.aconc + k_w A.aconc \frac{A.aconc^{n_4}}{K_4^{n_4} + A.aconc^{n_4}} \\
\frac{dB.aconc}{dt} &= k_3 B.iconc - \frac{k_4 A.aconc^{n_2}}{K_2^{n_2} + A.aconc^{n_2}} B.aconc \\
\frac{dB.iconc}{dt} &= -k_3 B.iconc + \frac{k_4 A.aconc^{n_2}}{K_2^{n_2} + A.aconc^{n_2}} B.aconc \\
\frac{dC.aconc}{dt} &= k_5 C.iconc - \frac{k_6 B.aconc^{n_3}}{K_3^{n_3} + B.aconc^{n_3}} C.aconc \\
\frac{dC.iconc}{dt} &= -k_5 C.iconc + \frac{k_6 B.aconc^{n_3}}{K_3^{n_3} + B.aconc^{n_3}} C.aconc
\end{aligned}$$

Model 5

$$\begin{aligned}
\frac{dA.aconc}{dt} &= k_1 A.iconc - \frac{k_2 C.aconc^{n_1}}{K_1^{n_1} + C.aconc^{n_1}} A.aconc - k_s A.aconc \frac{A.aconc^{n_4}}{K_4^{n_4} + A.aconc^{n_4}} \\
\frac{dA.iconc}{dt} &= -k_1 A.iconc + \frac{k_2 C.aconc^{n_1}}{K_1^{n_1} + C.aconc^{n_1}} A.aconc + k_s A.aconc \frac{A.aconc^{n_4}}{K_4^{n_4} + A.aconc^{n_4}} \\
\frac{dB.aconc}{dt} &= k_3 B.iconc - \frac{k_4 A.aconc^{n_2}}{K_2^{n_2} + A.aconc^{n_2}} B.aconc \\
\frac{dB.iconc}{dt} &= -k_3 B.iconc + \frac{k_4 A.aconc^{n_2}}{K_2^{n_2} + A.aconc^{n_2}} B.aconc \\
\frac{dC.aconc}{dt} &= k_5 C.iconc - \frac{k_6 B.aconc^{n_3}}{K_3^{n_3} + B.aconc^{n_3}} C.aconc \\
\frac{dC.iconc}{dt} &= -k_5 C.iconc + \frac{k_6 B.aconc^{n_3}}{K_3^{n_3} + B.aconc^{n_3}} C.aconc
\end{aligned}$$

3.3 Supplementary figures

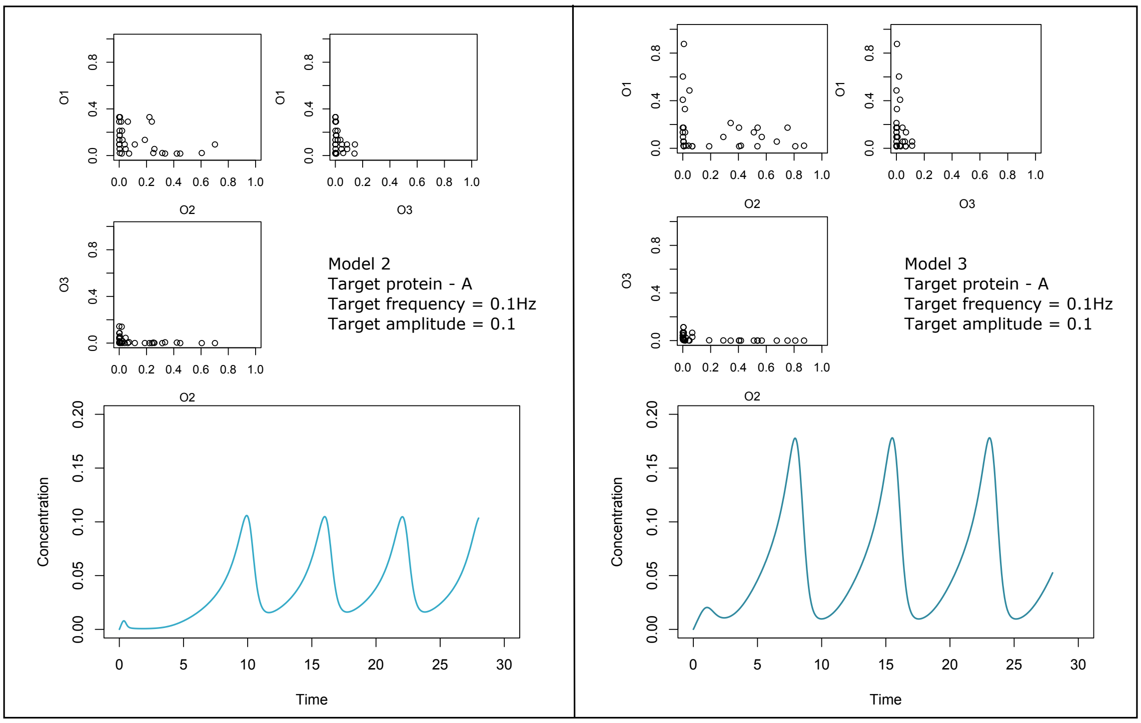

Figure S3: (top) Three two-dimensional projections of the Pareto front for the best performing models: (left) Model 2 and (right) Model 3, considering the behavior of the target active concentration of protein A described by a target frequency $f_t = 0.1Hz$ and a target amplitude $A_t = 0.1$. (bottom) Simulation of the active concentration of protein A using the structure of (left) Model 2 and (right) Model 3, and an arbitrarily selected set of optimal parameter values from the corresponding Pareto front (single point).

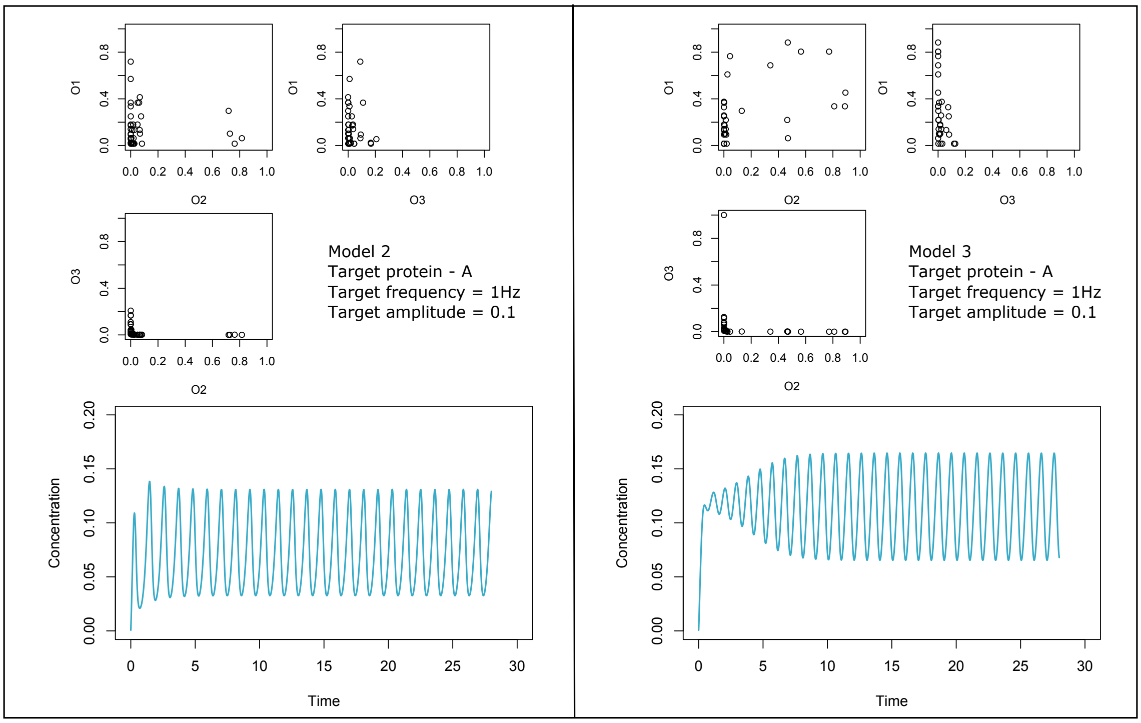

Figure S4: (top) Three two-dimensional projections of the Pareto front for the best performing models: (left) Model 2 and (right) Model 3, considering the behavior of the target active concentration of protein A described by a target frequency $f_t = 1Hz$ and a target amplitude $A_t = 0.1$. (bottom) Simulation of the active concentration of protein A using the structure of (left) Model 2 and (right) Model 3, and an arbitrarily selected set of optimal parameter values from the corresponding Pareto front (single point).

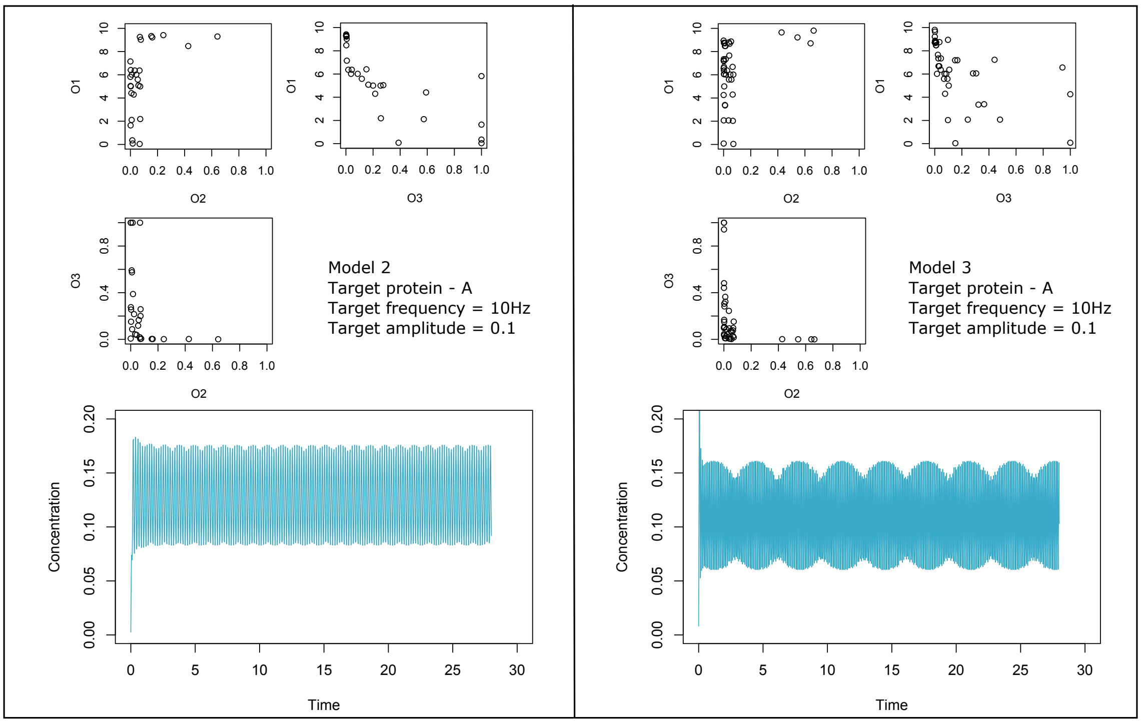

Figure S5: (top) Three two-dimensional projections of the Pareto front for the best performing models: (left) Model 2 and (right) Model 3, considering the behavior of the target active concentration of protein A described by a target frequency $f_t = 10Hz$ and a target amplitude $A_t = 0.1$. (bottom) Simulation of the active concentration of protein A using the structure of (left) Model 2 and (right) Model 3, and an arbitrarily selected set of optimal parameter values from the corresponding Pareto front (single point).

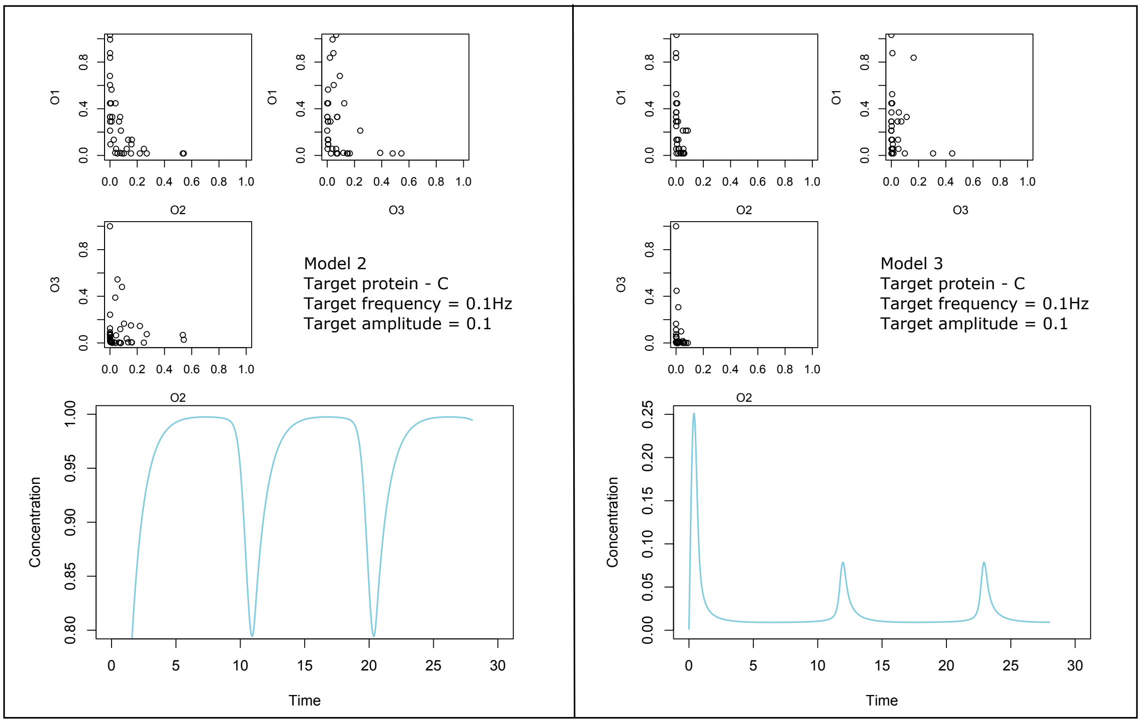

Figure S6: (top) Three two-dimensional projections of the Pareto front for the best performing models: (left) Model 2 and (right) Model 3, considering the behavior of the target active concentration of protein C described by a target frequency $f_t = 0.1Hz$ and a target amplitude $A_t = 0.1$. (bottom) Simulation of the active concentration of protein C using the structure of (left) Model 2 and (right) Model 3, and an arbitrarily selected set of optimal parameter values from the corresponding Pareto front (single point).

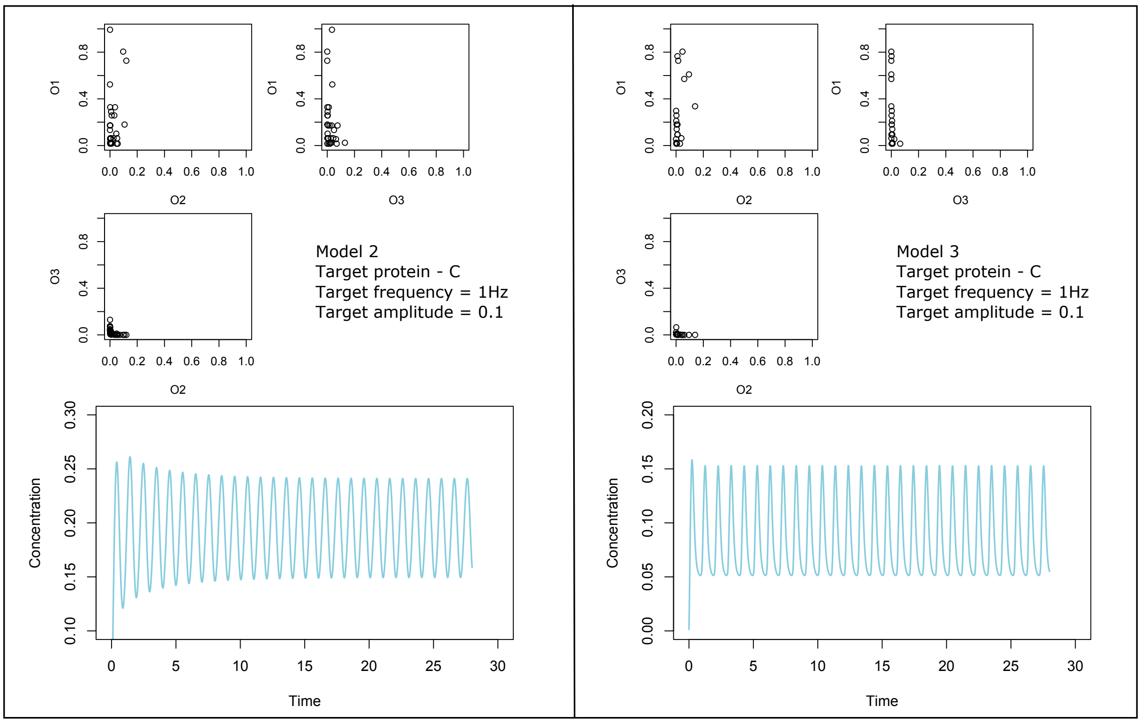

Figure S7: (top) Three two-dimensional projections of the Pareto front for the best performing models: (left) Model 2 and (right) Model 3, considering the behavior of the target active concentration of protein C described by a target frequency $f_t = 1Hz$ and a target amplitude $A_t = 0.1$. (bottom) Simulation of the active concentration of protein C using the structure of (left) Model 2 and (right) Model 3, and an arbitrarily selected set of optimal parameter values from the corresponding Pareto front (single point).

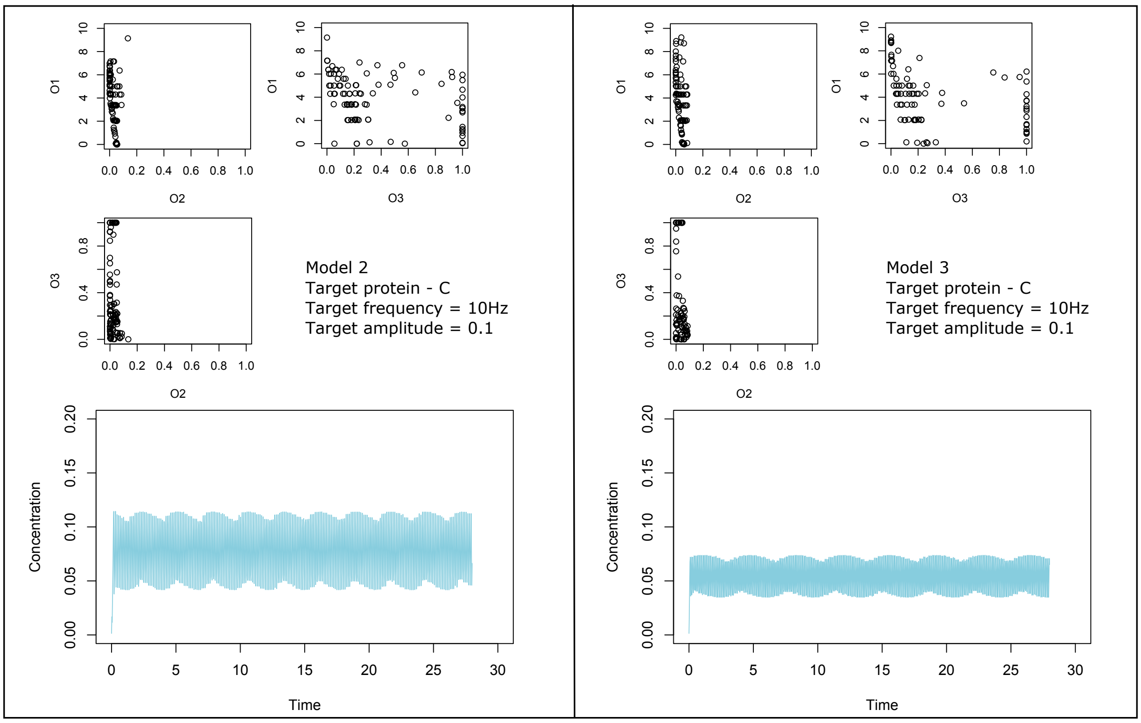

Figure S8: (top) Three two-dimensional projections of the Pareto front for the best performing models: (left) Model 2 and (right) Model 3, considering the behavior of the target active concentration of protein C described by a target frequency $f_t = 10Hz$ and a target amplitude $A_t = 0.1$. (bottom) Simulation of the active concentration of protein C using the structure of (left) Model 2 and (right) Model 3, and an arbitrarily selected set of optimal parameter values from the corresponding Pareto front (single point).
